# Intrinsic Entropy of Squeezed Quantum Fields and Nonequilibrium Quantum Dynamics of Cosmological Perturbations

**DOI:** 10.3390/e23111544

**Published:** 2021-11-20

**Authors:** Jen-Tsung Hsiang, Bei-Lok Hu

**Affiliations:** 1 Center for High Energy and High Field Physics, National Central University, Taoyuan 32001, Taiwan; 2 Maryland Center for Fundamental Physics and Joint Quantum Institute, University of Maryland, College Park, MD 20742, USA; blhu@umd.edu

**Keywords:** cosmological particle creation, entropy generation, nonequilibrium field theory, cosmological perturbations

## Abstract

Density contrasts in the universe are governed by scalar cosmological perturbations which, when expressed in terms of gauge-invariant variables, contain a classical component from scalar metric perturbations and a quantum component from inflaton field fluctuations. It has long been known that the effect of cosmological expansion on a quantum field amounts to squeezing. Thus, the entropy of cosmological perturbations can be studied by treating them in the framework of squeezed quantum systems. Entropy of a free quantum field is a seemingly simple yet subtle issue. In this paper, different from previous treatments, we tackle this issue with a fully developed nonequilibrium quantum field theory formalism for such systems. We compute the covariance matrix elements of the parametric quantum field and solve for the evolution of the density matrix elements and the Wigner functions, and, from them, derive the von Neumann entropy. We then show explicitly why the entropy for the squeezed yet closed system is zero, but is proportional to the particle number produced upon coarse-graining out the correlation between the particle pairs. We also construct the bridge between our quantum field-theoretic results and those using the probability distribution of classical stochastic fields by earlier authors, preserving some important quantum properties, such as entanglement and coherence, of the quantum field.

## 1. Introduction

Entropy of quantum cosmological perturbations is an important topic which has by now clocked almost three decades of investigations [1,2,3]. In terms of its theoretical foundation, it is built upon the bigger issue of (I) the entropy of quantum fields, where investigation started in the mid-1980s [4,5,6,7] continuing on into the 1990s [8,9,10,11,12], 2000s [13,14,15,16,17] and has been rekindled in recent years [18,19,20,21,22]. There are two aspects in this theme: (1) the quantum field theory component depicting particle creation from the vacuum and quantum cosmological perturbations; (2) the nonequilibrium statistical mechanics aspect describing the evolutionary dynamics of a quantum many-body system—the fluctuations in, and the dissipation of, a quantum field. These two components when combined make up the quantum field theory of nonequilibrium systems [23,24,25], the two major paradigms being the Boltzmann correlation hierarchy and the Langevin open systems [26]. These two components or aspects enter into many theoretical issues of fundamental interest such as quantum (de)coherence, quantum correlations and quantum entanglement. Concepts in open quantum systems [27,28,29] have been introduced, and advanced techniques in quantum field theory [7,23,24,25] have been utilized for these purposes [26]. Against this backdrop we wish to investigate the entropy of quantum fields and cosmological perturbations in a dynamical setting, such as in the early universe. Saving the discussions of the two theoretical factors for the next section, we focus here on the cosmological aspect; (II) The cosmological factor also has two components. They are: (1) gravitational perturbation theory which describes how weak classical perturbations of scalar (density contrast) vector (vorticity) and tensor (gravitational waves) types evolve in a dynamical spacetime; and (2) quantum matter field processes such as particle creation from vacuum fluctuations amplified by the expansion of the universe and their consequences.

### 1.1. The Cosmological Aspect: Gravitational Perturbations and Quantum Fluctuations

#### 1.1.1. Classical Gravitational Perturbations: Scalar, Vector and Tensor Components

Gravitational perturbation theory has been a well established subject in cosmological structure formation since the 1946 seminal paper of Lifshitz based on the amplification of density contrasts related to the (scalar sector of the) metric perturbations [30,31,32,33,34]. What seeded the structures in the classical gravitational perturbation theory was assumed to be from white noises. After the advent of the inflationary universe [35,36,37] where the vacuum energy density of a quantum scalar field, the inflaton, is believed to have driven the cosmos to (near-) exponential expansion for a certain duration in the early universe, one needs to take into account how the quantum scalar field and its fluctuations are coupled to the (scalar sector of the) metric perturbations controlling the density contrasts. If we regard Lifshitz’s 1946 paper as opening the first (classical) stage of cosmological structure formation investigation based on gravitational perturbation theory; this second (quantum) stage involving the inflaton’s fluctuations began in 1982 [38,39,40,41,42,43].

With the attention now focused on everything we see today in the universe as originating from quantum field mediated-gravitational perturbations, it is worth mentioning another important process involving fluctuations of quantum fields in the early universe, specifically, cosmological particle creation from the vacuum. The former class of activities placed in the inflationary universe context could take place as early as the GUT time ($10^{-35}$ s) while the latter happened even earlier, predominantly at the Planck time ($10^{-43}$ s).

#### 1.1.2. Quantum Field Processes Involving Vacuum Fluctuations

Quantum field processes in curved spacetime such as Hawking radiation from black holes or cosmological particle creation in the very early universe are described in great detail in many monographs, for example [44,45,46,47,48]. The amplitude of a classical wave mode can be parametrically amplified by a time-dependent drive [49]. It is the same way for vacuum fluctuations in a quantum field, resulting in the creation of particle pairs: the expansion of the universe acting like a drive, parametrically amplifying the quantum noise, giving rise to spontaneous particle production [49,50]. If there were particles already present in an initial state they will be amplified with the same amplification factor in stimulated production. This is like the spontaneous and stimulated emission of atoms in quantum optics. In fact, quantum field processes in a time-dependent background, be it in a cosmological spacetime or in an external laboratory field [51], can be captured by the ‘squeezing’ of quantum states [52,53,54,55]. The vacuum is ‘squeezed’ in the evolutionary history while particles are produced [56]. A summary description of cosmological particle creation in terms of squeezing can be found in, for example, [11,57,58].

We mention quantum cosmological perturbations and cosmological particle creation together because they are subjected to the same mechanism, which amplifies these perturbations or fluctuations, namely, as in parametric oscillators, where the frequencies of the normal modes are time-dependent. While parametrically amplified quantum fluctuations engender particle creation, parametrically amplified gravitational perturbations engender galaxies and structures, either classically with seeds of white noise or by the inflaton field’s quantum fluctuations.

#### 1.1.3. Distinguish Classical Perturbations from Quantum Fluctuations

It is of theoretical significance to make the distinction between classical linear gravitational perturbations, which are believed to be the progenitors of galaxies and structures we see today, and vacuum fluctuations of a quantum field, which engender the spontaneous creation of particle pairs, a subject fundamental to quantum field theory in curved spacetime. When we talk about the cosmological density contrasts, the isocurvature perturbations, the vorticity and the primordial gravitational waves, we are referring to quantities derived from the scalar, vector and tensor perturbations of the background spacetime. Density contrasts are derived from the scalar sector of the metric perturbations, a subject well explored in classical general relativity, culminating in Bardeen’s gauge invariant quantities [34,42]. In inflationary cosmology they are coupled to a quantum scalar field, the inflaton. To get one compact equation of motion for the density contrast, mixed metric perturbations + scalar field variables are used, such as the gauge invariant Mukhanov-Sasaki variable. This is all standard and fine. However, when one moves to the quantum theory of cosmological perturbations in inflationary universe, one has to be careful what this means, especially when dealing with decoherence and quantum to classical transition issues. The scalar (inflaton) field $\hat{\phi }\left(x,t\right)$ is intrinsically quantum in nature and comes with its quantum fluctuations. Often a background field expansion $\hat{\phi }\left(x,t\right)=\bar{\phi }\left(x,t\right)+\hat{\delta }\phi \left(x,t\right)$ is performed, assuming the background field $\bar{\phi }\left(x,t\right)$ is classical and the quantum character shows through its fluctuations $\hat{\delta }\phi \left(x,t\right)$. However, the gravitational perturbation component in this mixed variable is of classical origin. The scalar sector of the metric perturbations related to the Newtonian potential is a constraint, not a dynamical degree of freedom (the tensor modes, the gravitational waves, are). Its nature is determined by (or ‘slaved’ to [59,60]) the matter source. In general relativity when the matter source is classical, this scalar sector of the metric perturbation is classical. In inflationary cosmology, what determines the density contrast comes from both the classical scalar metric perturbations and the quantum inflaton field. When one says, ‘quantize the mixed variable’, one should bear in mind that the intrinsic nature of metric perturbations remains classical. In an extreme case, one may even conjure up situations where the quantum fluctuations of the scalar field are made to vanish, such as “choosing a (co-moving) gauge which for scalar perturbations makes the velocity perturbation vanish. For single field inflation, this means that the time coordinate is defined so that at any given time the scalar field equals its unperturbed value” (one is riding up and down with the scalar field’s fluctuations), “with all perturbations relegated to components of the metric” ([61], Section 5.3D). This does not mean that gravity has become quantum, only that the scalar perturbations now acquire a quantum nature by virtue of the presence of the inflaton field. Put it in another way, when there is no inflaton, one returns to purely classical general relativity. The Newtonian force is slaved to the source which is classical. There is no way for the gravitational perturbations to become quantum.

Now about the tensor sector. Gravity’s dynamical (or propagating) degrees of freedom reside only in the tensor sector, that is, the gravitational waves. Primordial gravitational waves are described by the tensor component of gravitational metric perturbations. They have also been studied at the classical level since 1946. One can consider quantizing the linearized tensor perturbations, whence they become the primordial gravitons (Note gravitational waves are weak metric perturbations, like sound waves, barring the differences between transverse and longitudinal waves. Gravitons are quantized linear perturbations of spacetime, like phonons, in the nature of collective excitations. This is a far cry from quantum gravity, defined as theories for the microscopic structures of spacetime at the Planck scale [62]). These gravitons still obey deterministic equations of motion. They are not stochastic intrinsically. Only when one considers a large number of primordial gravitons and use statistical means to describe their distributions would they become ‘stochastic’ (Probing further into their connection in cosmological perturbation theory Roura and Verdaguer [63] show that in the theoretical framework of stochastic gravity [45], at the Gaussian level, the stochastic variables give an equivalent description as the quantized linear perturbations [42]).

### 1.2. Related Issues: Decoherence and Entanglement

Before we delve into a full discussion of the entropy of cosmological perturbations and fluctuations we want to mention two issues in quantum information related to entropy, namely, decoherence and entanglement. This would be brief because each topic would merit a separate paper or two to describe.

(1) Decoherence of cosmological perturbations. Again, many questions are asked and different approaches have been suggested. To give two early examples, in Guth and Pi [64], by using an inverted harmonic oscillator model as an example and invoking the uncertainty relation they showed that the inflaton will, at late times, behave classically. Note that this is for a closed system thus, strictly speaking, it is at most dephasing, rather than decoherence. In Starobinsky’s stochastic inflation model [65], there are two key points: (a) the long wavelength perturbations are assumed to behave classically and (b) the short wavelength perturbations are treated as white noise. Under what conditions could (a) be realized and (b) be implemented? How could it be that a free field partitioned into two sectors in an expanding universe that the short wavelength sector would behave like white noise? One should also clarify the conditions this noise can effectively decohere the long wavelength sector by its backreaction. These questions were asked and challenged by, for example, [12,66]. This area of research begun in earnest in the early 1990s [12,67,68,69,70,71,72] is still being pursued vibrantly today. For a sample of recent work, see, for example, [21,22], where other earlier references can be found. We shall discuss this topic with a detailed background summary in a companion paper under preparation [73];

(2) Entanglement and entropy. The entanglement between particle pairs, one with momentum k, the other with −k, such as studied in [19,74] is relevant to our present consideration of entropy associated with particle creation. Another related topic is entanglement entropy. The seminal papers [75,76] explore the entropy of free quantum fields in Minkowski space with a partition, providing a more general and basic statistical mechanical way to understand black hole entropy. It was the precursor of the by-now familiar topic of entanglement entropy [77,78] hotly pursued in the last two decades. In cosmology when the spacetime is dynamical the natural ‘partition’ which separates modes into two sectors is the Hubble horizon defined by the inverse of the expansion rate. Entanglement between two Unruh–DeWitt detectors has been studied concerning the effects of spacetime curvature [79,80,81,82] and topology [83] and under other conditions in the emergent field of relativistic quantum information [84], but that is not the main concern in our present study. We now turn to the two aspects mentioned in the beginning, that of quantum field theory and nonequilibrium dynamics.

## 2. Entropy of Quantum Fields

Defining and understanding entropy for quantum fields is a task of fundamental significance in both quantum field theory and nonequilibrium statistical mechanics. For free fields in a dynamical setting (driven by some external source or in a dynamical spacetime as in cosmology) with no spatial boundary or event horizon present (thus not involving entanglement entropy) the authors of [4] provided a first answer to the following question: Is there entropy production in particle creation from the vacuum? Adhering to quantum field theory, because the vacuum is a pure state and particle creation is a unitary process, the answer should be no. However, even in textbooks one sees that an entropy, say, of photons, can be assigned proportionally to the number of particles present. These two seemingly contradictory answers each seem to stand on its firm ground. How does one reason out their differences? This paradox was what lured one of us into exploring this issue and came out with some interesting discoveries. The first observation is: the first answer seems to focus on the initial state while the second answer on the final outcome. For the second answer to make sense some information must have been lost. Two possibilities come to one’s mind. Either (a) some essential information is ignored, implicitly when one defines the field entropy in terms of particle numbers, or (b) some kind of coarse-graining measure is introduced explicitly which curtails some information of the system.

For (a), when one argues that the entropy of a free quantum field is proportional to the particles created, one often implicitly adopts a Fock space representation to capture the particle number, while ignoring completely the information about the correlation and the coherence in the particle pairs. This was pointed out in [4]. Note that the entropy of a closed system is defined by taking the full trace, which is independent of the basis or representation, and there is no priori definition or requirement that the entropy be a function of the particle number. This point is illustrated explicitly in [85] where an equation governing the quantum coherence is presented alongside that governing the particle numbers, from which the entropy is calculated. For (b), in a realistic setting, after particles are created from the vacuum, they interact, and information about their interaction could be lost, e.g., if one focuses on the scattering cross section but ignores some of their correlations. This second point is illustrated in an interacting harmonic oscillator model of field theory in [5] and later with greater depth in [14], which proves an *H*-theorem in such coarse-grained systems.

### 2.1. Intrinsic Entropy of Free Quantum Fields

Entropy of quantum fields is a fundamental, seemingly simple, yet somewhat tricky issue. Early conceptual inquiries [4,5] made explicit the specific underlying conditions which allow for the entropy of free quantum fields to be related to particle creation numbers (for boson fields). Namely, by adopting a Fock space representation and making statements only in terms of number operators, one implicitly ignores all quantum phase information which determines the coherence (See [85] for the interplay of both quantities). From this, it is easy to understand why the entropy associated with particle production is proportional to the degree of squeezing, in cosmology by the expansion of the universe [3,8,9,10,86], or more down to earth, the moving mirror in the dynamical Casimir effect [87].

### 2.2. Entropy of Interacting Quantum Fields

We have alluded to this class of theories above, the simplest exemplified by the anharmonic oscillator models in quantum mechanics. A more systematic analysis is based on the Boltzmann paradigm applied to the BBGKY hierarchy, namely, the ‘slaving’ of the higher order correlation functions to the lower ones, with Boltzmann equation being the lowest order, describing the dynamics of the one point distribution function with source from the two particle collision integral. The assumption of a causal factorizable initial condition on the two-particle correlation function gives rise to a dissipative dynamics in the one particle distribution function. For interacting quantum field, it is the concept of *correlation entropy* defined in the Schwinger–Dyson hierarchy for a certain *n*th order correlation functions (see [13,14,88,89]). Notice the fundamental difference between the ‘truncation’ and the ‘slaving’ procedures, the former giving rise to a unitary equation such as for the mean field in the Vlasov equation, the latter a (nonunitary) dissipative Boltzmann equation.

### 2.3. Entropy Measures

As we stated in the beginning and as is well known, entropy arises from a loss of information, either by choice or by necessity. Thus, any mention of entropy measure must be accompanied by the specification of what information is being dropped, lost, or operationally inaccessible. That, in turn, depends on one’s coarse-graining choices or one’s level of ignorance. We give as an example two commonly used coarse-graining measures.

#### 2.3.1. Coarse-Graining by Discrepancies of Scales

If the relevant physical variables in a system have clear discrepancies in scales, be it slow vs. fast, low vs. high frequency, light vs. heavy mass, one can decide based on physical conditions which scale is of the most physical interest and proceed to coarse-grain the other variables, one level at a time in an orderly nested way. Famous examples are the slow variables of van Hove, the adiabatic invariants for a parametric process, the Born-Oppenheimer approximation wherein the heavy mass (e.g., nucleus) motion is considered as effectively static with respect to the light mass (e.g., electron), etc. Formally, these situations are best described by the projection operator formalism of Zwanzig-Mori-Nakajima [90]. In quantum systems, one measure often adopted is the coarse-graining of the phase information since the phase often varies rapidly. This is behind the random phase approximation. The rotating wave approximation (RWA) is one of the two preambles of quantum statistical mechanics, where the density matrix of a quantum system is assumed to be diagonal. By eliminating all phase information one can consider only probabilities, which is why quantum statistical mechanics taught from textbooks misses all the quantum coherence and entanglement issues.

#### 2.3.2. Coarse-Graining by Partitioning

If there is a clear cut partition in the system one is interested in, one can use it to separate the variables of special interest from other variables perhaps of lesser interest. In the stochastic inflation example, the horizon is the divider between the long wavelength sector and the short wavelenght sector. The physical issues of concern are accordingly different: for the former, decoherence—how could they be viewed as classical? For the latter, noise—do they really constitute white noise? For interacting quantum fields, if there is only one field and one has chosen the appropriate partition one can invoke the coarse-grained effective action (CGEA) [91,92,93,94] to describe the backreaction effects of the coarse-grained sector viewed as the environment to the system. The ‘in–in’, Schwinger–Keldysh, or ‘closed-time-path’ version of CGEA is equivalent to the Feynman–Vernon influence functional formalism. This approach was used to describe cosmological decoherence in [12,68,69,72]. If there is more than one field, it is even easier, because the system field and the environment field are distinct and their coupling does not change in time. One can decide which field should be considered as one’s system and coarse-grain the others [20,95,96,97]. One does not need to keep track of the time-varying partition in an expanding universe.

In cosmology, when the spacetime is dynamical, the natural ‘partition’ is between the modes of wavelengths greater or less than the horizon, defined by the inverse of the Hubble expansion rate—between the super and sub horizon sectors. This is referred to as entanglement entropy in cosmology. Recent work includes [22,98,99,100,101], where earlier references can be found.

### 2.4. Approaches Taken Here in Relation to Earlier Work

From the pioneering work in the 1980s and 1990s mentioned above, it is encouraging to see that the conceptual framework of open quantum systems and the technical tools of nonequilibrium quantum field theory are more widely adopted in current theoretical cosmological research. For recent work see, for example, [20,21,22,97,100,101,102] and references therein. We now focus on the specific main points on the entropy of cosmological perturbations, analyzed here from a squeezed open quantum system perspective using techniques from nonequilibrium quantum field theory and relate them to earlier work.

(1) The theoretical framework useful for our purpose is known as ‘squeezed open quantum systems’ [57,58,86] with cosmology being an important exemplary category [11]. For the entropy measure we shall follow:

(A) the vein of [4,85] where, using a Fock basis measuring the number of particles created, or the cosmological perturbations, we show explicitly when the quantum correlations between the particle pairs are kept, there is no entropy generation, whereas when the correlation is ignored, we obtain the well known expression for the entropy proportional to the number of particles. Related to [85] is the work of [17] where the entropy is calculated from the von Neumann entropy constructed from the reduced density matrix defined with respect to a second adiabatic order number state (Of interest is their conclusion that there is no decoherence as claimed in [103], despite their ‘cute’ way of explaining why there is decoherence);

(B) Our demonstration that there is entropy generated by coarse-graining the −k component of the particle pair corroborates the results obtained by [74] via a different pathway, by showing the destruction of the entanglement between the particle pair.

(2) The formal structure. Many authors, including those of the major work [1,2] (BMP) and [19] (CP) on the entropy of cosmological perturbations, correctly start with the formal structure of an interacting quantum field theory. Treating interacting fields in the framework of the classical BBGKY or the quantum Schwinger–Dyson hierarchy—the Boltzmann paradigm—is a serious challenge. It was taken up in earnest in the work of [12,13,14,88], developing the concepts of correlation histories, correlation dynamics and correlation noise. Their master effective action and the way how the higher order correlators should be ‘slaved’ to a lower order correlator provide the basis for formulating effective field theories for open quantum systems [104], an important theme which saw fruitful applications to cosmological issues in [20,97]. In the same vein, entropy from non-Gaussian states is pursued by [18,105]. However, BMP and CP quickly assume a Gaussian truncation of the BBGKY or the Schwinger–Dyson hierarchy, thus restricting their attention only to the lowest two-point functions. This is enough to treat linear perturbations and effectively places their theories on the same footing as free fields, where considerations in [4,74] would suffice. CP refer to the reduced density matrices defined by truncating the hierarchy at the first level as Gaussian and homogeneous density matrices (GHDM). Coarse-graining the phase information is also used in two major works: In [1,2], their coarse-grained entropy is obtained by averaging the two point correlation function over the phases. In [19] the growth of entropy associated with the coarse-graining is described by the set of Gaussian two-mode reduced density matrices $\rho_{\left(+k,-k\right)}^{\mathrm{red}}$, each characterizes the loss of entanglement between the two modes in the presence of interactions.

This is also the starting point of the way BMP defines entropy for the gravitational field. However, instead of treating a quantized field, they consider a classical stochastic field. Thus their considerations are predicated upon the quantum perturbations having effectively decohered: “Provided there is decoherence and $\sinh r_{k}\gg 1$ (where $r_{k}$ is the squeeze parameter of the kth mode), then we can take the classical limit where the classical correlation functions can be identified with quantum expectation values, i.e., $\langle \phi \left(x\right)\phi \left(y\right)\rangle =\langle 0|\hat{\phi }\left(x\right)\hat{\phi }\left(y\right)|0\rangle$”. Note the angular brackets on the lefthand side denotes a statistical average. The decoherence procedure is clearly stated in [3] as dropping the off-diagonal components of the density matrix. At the time these papers were written decoherence as an important theoretical issue was not that well understood. See more recent papers, such as [18,22,73,105], where the developments in the last 30 years can be traced back. We have devoted a subsection at the end of this paper to clarify the theoretical differences between a quantum field theory treatment and a classical stochastic field theory treatment albeit the two can serve some common purposes.

### 2.5. Our Findings and Organization

Our narrative takes on four steps, showing: (1) that the cosmological expansion results in the squeezing of the quantum field; (2) the nonequilibrium dynamics of a squeezed quantum field as a closed system, seen through the evolution of the density matrix operator in terms of the covariance matrix elements; (3) that the von Neumann entropy is zero in this closed system, as expected, and proportional to the particle numbers when the quantum correlation between the particle pair is coarse-grained away. We derive (4) the Wigner function of the quantum Gaussian field driven by a parametric process, which gives a clear picture of how the coarse-grained entropy may emerge, and whose phase-space description leads to the quantum thermodynamics of this system. With this, we also discuss (5) the differences and commonalities between our quantum field-theoretical methodology and approaches in the literature using the probability distribution of classical stochastic fields, especially on the ‘uniquely quantum’ features such as decoherence and entanglement issues. While facts in (1) and (3) are largely known before, we try to capture a global perspective and point out the subtleties in the meaning of entropy and how it is defined in closed quantum systems. Points (2), (4), and (5) form the technical core and the results we obtained are new.

This paper is organized as follows: in Section 3, to be self-contained, we provide a quick overview of the essential ingredients of first-order classical cosmological perturbation theory, and link the cosmological perturbations to the classical parametric field via the Mukhanov–Sasaki variables. In Section 4 we formally compute the covariance matrix elements of the parametric field as the basic building blocks of a Gaussian system. Then in Section 5, we decompose the field into a collection of parametric oscillators to take advantage of the oft-used nonequilibrium formalism to construct the density matrix operator and the density matrix elements. Doing so equips us to tackle the full dynamical evolution of the physical observables and derive the nonequilibrium thermodynamics of the system under study. We present the time evolution of the density matrix and its elements in Section 6, where we combine the language of the Bogoliubov transformation and the aforementioned nonequilibrium formalism for a succinct description of the full dynamics. In Section 7, we show entropy production associated with the particle pairs in the parametric development of the cosmological perturbations. In Section 8, we construct the Wigner function of the quantum field and show how it evolves in time. As a bonus we also show the relation between our quantum field theoretic results and those using probability distribution of classical stochastic fields by earlier authors. We point out that stochastic cannot be a complete replacement of quantum, especially over the core quantum issues like entanglement and quantum coherence of the field.

## 3. Cosmological Perturbation Theory in a Nutshell

In this short section we give a brief summary of cosmological perturbation theory [42,106] to make our presentation self-contained and to define the notations, which have evolved to become quite standardized over the years. Readers familiar with this topic can skip to the next section.

### 3.1. Gauge Transformations

The linear perturbations $\delta g_{\mu \nu }$ off a background spacetime with metric ${\bar{g}}_{\mu \nu }$. In the case of a Friedmann-Lemaître-Robertson-Walker (FLRW) universe background with scale factor *a* we can write the metric as:

(1) $$g_{\mu \nu }={\bar{g}}_{\mu \nu }+\delta g_{\mu \nu }=a^{2}\left(\gamma_{\mu \nu }+h_{\mu \nu }\right)\;,$$

where $h_{\mu \nu }$ and its derivatives are assumed to be small compared to the background metric $\gamma_{\mu \nu }$, which is used for the raising and lowering of the indices for the metric perturbations. Namely,

(2) $$\begin{array}{cccc} h^{\mu }{}_{\nu } & =\gamma^{\mu \alpha }h_{\alpha \nu }\;,\;\;\;\;\; & h^{\mu \nu } & =\gamma^{\mu \alpha }\gamma^{\nu \beta }h_{\alpha \beta }\;,\;\; \end{array}$$

such that the inverse metric of the perturbed universe is:

(3) $$g^{\mu \nu }=a^{-2}\left(\gamma^{\mu \nu }-h^{\mu \nu }\right)\;.$$

The metric perturbations $h_{\mu \nu }$ can be decomposed into the scalar, vector and tensor components, but the scalar component of the perturbations is essential for the consideration of cosmological density contrasts. The line element can therefore be parametrized as:

(4) $$ds^{2}=a^{2}\left(\eta \right)\left\{-\left(1+2A\right)\;d\eta^{2}-2B_{,\;i}\;d\eta dx^{i}+\left[\left(1-2\psi \right)\;\delta_{ij}+2\partial_{i}\partial_{j}E\right]\;dx^{i}dx^{j}\right\}\;,$$

by four scalars A, B, ψ and E. Here, η is the conformal time coordinate and $x^{i}$ are the spatial coordinates and we assume the background universe is spatially flat.

Now consider the choice of gauge and the construction of gauge-invariant variables. The infinitesimal coordinate transformation between two coordinate systems, say $\left\{x^{\alpha }\right\}$ and $\left\{{\tilde{x}}^{\alpha }\right\}$, in the perturbed spacetime is given by:

(5) $${\tilde{x}}^{\alpha }=x^{\alpha }+\xi^{\alpha }\;,$$

where $\xi^{\alpha }$ and their derivatives are first-order small in *h*. It then can be shown that the four scalars in the line element will transform as:

(6) $$\begin{array}{cccccc} \tilde{A} & =A-\xi^{0}{}^{\prime }-\frac{a^{\prime }}{a}\;\xi^{0}\;,\; & \tilde{\psi } & =\psi +\frac{a^{\prime }}{a}\;\xi^{0}\;,\; & \tilde{B}-{\tilde{E}}^{\prime } & =B-E^{\prime }+\xi^{0}\;, \end{array}$$

where the prime denotes the derivative with respect to the conformal time, and ξ is the scalar component of the spatial vector $\xi^{i}$. Among them, we can form two gauge-invariant Bardeen potentials:

(7) $$\begin{array}{cccc} \Phi & =A+\frac{a^{\prime }}{a}\left(B-E^{\prime }\right)+{\left(B-E^{\prime }\right)}^{\prime }\;,\;\; & \Psi & =\psi -\frac{a^{\prime }}{a}\left(B-E^{\prime }\right)\;,\; \end{array}$$

which are invariant under the coordinate transformation (5), and $\tilde{\Phi }=\Phi$ and $\tilde{\Psi }=\Psi$. If we choose ξ=−E and $\xi^{0}=-\left(B-E^{\prime }\right)$, then we have $\tilde{B}=0$ and $\tilde{E}=0$. This is the Newtonian gauge, in which $\tilde{A}=\tilde{\Phi }$, and $\tilde{\psi }=\tilde{\Psi }$ become gauge-invariant. In terms of the gauge-invariant quantities the line element reduces to:

(8) $$ds^{2}=a^{2}\left\{-\left(1+2\Phi \right)\;d\eta^{2}+\left(1-2\Psi \right)\delta_{ij}\;dx^{i}dx^{j}\right\}\;.$$

The Bardeen potentials will be equal if the stress tensor of matter has no anisotropic perturbations.

### 3.2. Scalar Field Perturbations

Next we turn to the perturbation of a real scalar field (We may consider non-minimal coupling: adding a term proportional to the scalar curvature for a spatially-flat spacetime would amount to an additional term in the time-dependent frequency of the modes. This is discussed in e.g., Section 2.1.4 of [45]. If the ‘non-minimal’ coupling is conformal coupling, then under a conformal transformation of the field, in the conformal time, one gets an equation of the same form as a quantum field in flat space with time-dependent ‘effective mass’, as is studied in (21)).

(9) $$S_{\phi }=-\int \;d^{4}x\sqrt{-g}\;\;\left\{\frac{1}{2}\;g^{\mu \nu }\partial_{\mu }\phi \partial_{\nu }\phi +V\left(\phi \right)\right\}\;,$$

where *V* is its potential. We shall use a background field expansion $\phi =\bar{\phi }+\delta \phi$ around a background field $\bar{\phi }$ and focus on the perturbations δϕ. We will assume that the unperturbed spacetime is homogeneous and isotropic, and that the background field $\bar{\phi }\left(\eta \right)$ in this spacetime is a function of time only. The scalar field perturbations δϕ(x,η) obey the wave equation:

(10) $$\delta \phi^{\prime \prime }-\partial^{2}\delta \phi +2H\delta \phi^{\prime }+a^{2}\;\frac{\partial^{2}V\left(\bar{\phi }\right)}{\partial {\bar{\phi }}^{2}}\;\delta \phi =\left(A^{\prime }+3D^{\prime }+\partial^{2}B\right){\bar{\phi }}^{\prime }-2a^{2}A\;\frac{\partial V(\bar{\phi })}{\partial \bar{\phi }}\;,$$

where $H=a^{\prime }/a$ is the Hubble expansion rate in conformal time, and $\partial^{2}$ denotes $\sum_{i}\partial_{i}^{2}$. We see that the scalar field perturbations couple only to scalar metric perturbations.

After some algebra, one can derive the Einstein equations for the scalar perturbations given by:

(11) $$\begin{array}{cc} \partial^{2}\Phi -3H\Phi^{\prime }-3H^{2}\Phi & =4\pi a^{2}\left[\frac{1}{a^{2}}\left({\bar{\phi }}^{\prime }\;\delta \phi^{\prime }-{\bar{\phi }}^{\prime 2}\Phi \right)+\frac{\partial V(\bar{\phi })}{\partial \bar{\phi }}\;\delta \phi \right]\;,\; \end{array}$$

(12) $$\begin{array}{cc} \Phi^{\prime }+H\Phi & =4\pi {\bar{\phi }}^{\prime }\delta \phi \;,\; \end{array}$$

(13) $$\begin{array}{cc} \Phi^{\prime \prime }+3H\Phi^{\prime }+\left(2H^{\prime }+H^{2}\right)\Phi & =4\pi a^{2}\left[\frac{1}{a^{2}}\left({\bar{\phi }}^{\prime }\;\delta \phi^{\prime }-{\bar{\phi }}^{\prime 2}\Phi \right)-\frac{\partial V(\bar{\phi })}{\partial \bar{\phi }}\;\delta \phi \right]\;,\; \end{array}$$

with the background field $\bar{\phi }$ satisfying:

(14) $${\bar{\phi }}^{\prime \prime }+2H{\bar{\phi }}^{\prime }+a^{2}\frac{\partial V(\bar{\phi })}{\partial \bar{\phi }}=0\;.$$

Recall that Φ is the Bardeen potential. Taking a suitable superposition of the metric perturbation and the scalar field perturbation, Mukhanov and Sasaki define the gauge-invariant variable *u* in the conformal Newtonian gauge as:

(15) $$u=\frac{a^{2}}{a^{\prime }}\;{\bar{\phi }}^{\prime }\left(\Phi +\frac{a^{\prime }}{a{\bar{\phi }}^{\prime }}\delta \phi \right)\;$$

and rewrite the Einstein equation in the conformal Newtonian gauge into the Mukhanov–Sasaki equation:

(16) $$\begin{array}{cccccc} u^{\prime \prime }-\partial^{2}u-\frac{z^{\prime \prime }}{z}\;u & =0\;,\; & \; & \mathrm{with}\;\;\; & z & =\frac{a^{2}}{a^{\prime }}\;{\bar{\phi }}^{\prime }\;. \end{array}$$

With the scalar field playing the role of the inflaton, in the slow-roll limit, the spacetime is nearly de Sitter and (16) will assume the form

(17) $$u^{\prime \prime }-\partial^{2}u-H^{2}\left(2+5\epsilon -3\eta \right)\;u=0\;,$$

where the slow-roll parameters ϵ and η are given by:

(18) $$\begin{array}{cccc} \epsilon & =\frac{m_{p}^{2}}{2}\;\frac{V^{\prime 2}}{V^{2}}\;,\;\;\;\;\;\;\; & \eta & =m_{p}^{2}\;\frac{V^{\prime \prime }}{V}\;,\; \end{array}$$

with the Planck mass $m_{p}^{2}=1/8\pi$. Alternatively, we may rewrite (16) to obtain the wave equation of the curvature perturbation:

(19) $$R^{\prime \prime }-\partial^{2}R+2H\left(1+2\epsilon -\eta \right)R^{\prime }=0\;,$$

in the slow-roll limit, where the curvature perturbation R is related to the Mukhanov-Sasaki variable *u* by u=−zR. We observe both Equation (16) and (19) are in the form of an equation of motion for a parametric field u(x,η) or R(x,η) in flat space. The gravitational influence is wrapped in the function of z(η) which, according to (16), contains the effects of the scale factor or the inflaton potential $V(\bar{\phi })$. Thus, for our purpose here, it suffices to focus on this equation and discuss the dynamics of a generic parametrically-driven quantum scalar field in flat space as we shall do in the next sections.

## 4. Dynamics of a Parametrically-Driven Quantum Field

We consider a real scalar parametric field ϕ(x,t) in a flat spacetime, whose Lagrangian density is given by:

(20) $$L=-\frac{1}{2}\left[\eta^{\mu \nu }\partial_{\mu }\phi \partial_{\nu }\phi +m^{2}\left(t\right)\phi^{2}\right]\;,$$

with the Minkowski metric $\eta^{\mu \nu }$. We use the convention with signature (−1,+1,+1,+1). The time-dependent function $m^{2}\left(t\right)$ will effectively account for the parametric processes from the external agent. The corresponding equation of motion is:

(21) $$-\partial_{\mu }\left(\eta^{\mu \nu }\partial_{\nu }\phi \right)+m^{2}\left(t\right)\phi =0\;.$$

Expanding the field variable ϕ in terms of the mode function $\phi_{k}\left(t\right)$ of the form

(22) $$\begin{array}{cccccc} \phi (x,t) & =\sum_{k}e^{+ik\cdot x}\phi_{k}\left(t\right)\;, & \; & \mathrm{with}\;\;\; & \phi_{+k}^{*}\left(t\right) & =\phi_{-k}\left(t\right)\;, \end{array}$$

we find the action given by:

(23) S=−12∫dt∑k−ϕ˙k∗ϕ˙k∗+k2+m2ϕk∗ϕk∗.

Since ϕ˙+k∗ϕ˙+k∗=ϕ˙−k∗ϕ˙−k∗ and the summand in (23) takes the same form for ±k, we can write the action only in terms of the k>0 modes:

(24) S=∫dtL(ϕk,ϕ˙k)=−∫dt∑k>0−ϕ˙k∗ϕ˙k∗+k2+m2ϕk∗ϕk∗.

With the momentum conjugate to $\phi_{k}$ defined by:

(25) $$\pi_{k}=\frac{\partial L}{\partial {\dot{\phi }}_{k}}={\dot{\phi }}_{k}^{*}\;,$$

the Euler–Lagrange equation gives the equation of motion for $\phi_{k}\left(t\right)$

(26) $${\ddot{\phi }}_{k}+\left(k^{2}+m^{2}\right)\phi_{k}=0\;.$$

For the treatment of quantum fields we promote the field to be and operator. In the Heisenberg picture the expectation value will be computed with respect to the initial state. Then for each mode k we have:

(27) ϕ^+k†(t)=ϕ^−k†(t),      π^k=ϕ^˙k†=ϕ^˙−k†.

On the other hand, since the momentum $\hat{\pi }$ conjugate to the field operator $\hat{\phi }$ is given by $\hat{\pi }=\dot{\hat{\phi }}$, we have:

(28) π^=ϕ^˙=∑kϕ^˙ke+ik·x=∑kϕ^˙−k†e−ik·x=∑kϕ^˙k†e−ik·x=∑kπ^ke−ik·x.

Thus, the Fourier expansion of the momentum operator $\hat{\pi }$ takes a different form from that of $\hat{\phi }$ in (22). This is necessary to ensure the standard form of the equal-time canonical commutation relation:

(29) $$\left[\hat{\phi }\left(x,t\right),\hat{\pi }\left(x^{\prime },t\right)\right]=\sum_{k,k^{\prime }}\left[{\hat{\phi }}_{k}\left(t\right),{\hat{\pi }}_{k^{\prime }}\left(t\right)\right]\;e^{ik\cdot x-ik^{\prime }\cdot x^{\prime }}=\sum_{k}e^{ik\cdot (x-x^{\prime })}=i\;\delta^{\left(3\right)}\left(x-x^{\prime }\right)\;,$$

if we require:

(30) $$\left[{\hat{\phi }}_{k}\left(t\right),{\hat{\pi }}_{k^{\prime }}\left(t\right)\right]=i\;\delta_{k,k^{\prime }}\;.$$

In the general case, (27) allows an expansion:

(31) ϕ^k(t)=a^+k†uω∗(t)+a^−k†uω∗(t),and  π^k(t)=a^−k†u˙ω∗(t)+a^+k†u˙ω∗(t).

Here, $u_{\omega }\left(t\right)$ is a solution of (26), satisfying the positive frequency condition at a specified initial time with $\omega^{2}\left(t\right)=k^{2}+m^{2}\left(t\right)$, and a^k†, ${\hat{a}}_{k}^{†}$ are the annihilation and creation operators associated with $u_{\omega }$ at the initial time, satisfying the usual commutation relations:

(32) a^k†,a^k′†=δkk′,

and zero otherwise. Then the commutation relation (30) is consistently obeyed due to the Wronskian condition of $u_{\omega }$:

(33) uω∗(t)u˙ω∗(t)−uω∗(t)u˙ω∗(t)=i.

We may invert (31) to express ${\hat{a}}_{k}$ in terms of ${\hat{\phi }}_{k}$ and ${\hat{\pi }}_{k}$,

(34) a^k=−iu˙ω∗ϕ^+k−uω∗π^−k∗,

and confirm the commutation relations (32) for the creation and the annihilation operators.

From the expansions (31), we may compute the corresponding correlation or covariance matrix elements among various modes [107]:

(35) 12〈ϕ^k,ϕ^k′〉=12〈a^+k†,a^+k′†〉uω∗uω′∗+12〈a^−k†,a^+k′†〉uω∗uω′∗+12〈a^+k†,a^−k′†〉uω∗uω′∗+12〈a^−k†,a^−k′†〉uω∗uω′∗,

(36) 12〈ϕ^k†,ϕ^k′†〉=12〈a^+k†,a^+k′†〉uω∗uω′∗+12〈a^−k†,a^+k′†〉uω∗uω′∗+12〈a^+k†,a^−k′†〉uω∗uω′∗+12〈a^−k†,a^−k′†〉uω∗uω′∗,

(37) 12〈ϕ^k†,π^k′†〉=12〈a^+k†,a^+k′†〉uω∗u˙ω′∗+12〈a^−k†,a^+k′†〉uω∗u˙ω′∗+12〈a^+k†,a^−k′†〉uω∗u˙ω′∗+12〈a^−k†,a^−k′†〉uω∗u˙ω′∗.

The results for $\langle \left\{{\hat{\pi }}_{k},\;{\hat{\pi }}_{k^{\prime }}\right\}\rangle$ will be obtained by replacing $u_{\omega }$ in (35) by ${\dot{u}}_{\omega }$. These elements are extremely useful in constructing physical observables of a Gaussian system in that, for each mode, we can simplify calculations involving an infinite-dimensional density matrix operator into those based a 2×2 covariance matrix.

If the state used to evaluate the expectation value is a stationary state, that is, $\langle {\hat{a}}_{k}^{2}\rangle =0$, then (35)–(37) reduce to:

(38) 12〈ϕ^k,ϕ^k′〉=δ+k,−k′〈N^+k〉+12+〈N^−k〉+12uω∗uω∗,

(39) 12〈ϕ^k†,ϕ^k′†〉=δ+k,+k′〈N^+k〉+12+〈N^−k〉+12uω∗uω∗,

(40) 12〈ϕ^k†,π^k′†〉=δ+k,+k′〈N^+k〉+12uω∗u˙ω∗+〈N^−k〉+12u˙ω∗uω∗,

where N^k=a^k†a^k† is the number operator of mode k. Here we see that both ±k modes contribute. Equations (38)–(40) imply that

(41) 〈ϕ^±k2(t)〉=0,〈ϕ^±k†(t)π^±k†(t)〉=0.

These ingredients will allow us to construct the density matrix operator of the field, which will be used to compute the expectation values or observables of the field.

## 5. Nonequilibrium Evolution of the Density Matrix

We now proceed to construct the density matrix operator of the quantum parametric scalar field using the formalisms developed in [108]. We begin by writing the field mode operators in the form of harmonic oscillators.

As the operators ${\hat{\phi }}_{k}$, ${\hat{\pi }}_{k}$ are not Hermitian, we decompose them into the real and the imaginary parts by:

(42) $$\begin{array}{cc} {\hat{\phi }}_{k} & =\frac{1}{\sqrt{2}}\;\left({\hat{\phi }}_{k}^{\left(1\right)}+i\;{\hat{\phi }}_{k}^{\left(2\right)}\right)\;. \end{array}$$

Hence the action (24) becomes:

(43) $$\begin{array}{c} S=\frac{1}{2}\int \;dt\;\sum_{k>0}\left[\left({\dot{\phi }}_{k}^{(1)2}+{\dot{\phi }}_{k}^{(2)2}\right)-\omega^{2}\left(t\right)\left(\phi_{k}^{(1)2}+\phi_{k}^{(2)2}\right)\right]\;. \end{array}$$

with the real and the imaginary parts acting as uncoupled parametric oscillators and $\omega^{2}\left(t\right)=k^{2}+m^{2}\left(t\right)$.

The corresponding conjugate momenta are given by:

(44) $$\begin{array}{cccc} p_{k}^{\left(1\right)} & =\frac{\partial L}{\partial {\dot{\phi }}_{k}^{\left(1\right)}}={\dot{\phi }}_{k}^{\left(1\right)}\;,\;\;\;\; & p_{k}^{\left(2\right)} & =\frac{\partial L}{\partial {\dot{\phi }}_{k}^{\left(2\right)}}={\dot{\phi }}_{k}^{\left(2\right)}\;, \end{array}$$

which implies

(45) $${\hat{\pi }}_{k}={\dot{\hat{\phi }}}_{k}^{†}=\frac{1}{\sqrt{2}}\left(p_{k}^{\left(1\right)}-i\;p_{k}^{\left(2\right)}\right)\;.$$

Note the sign before the imaginary part of ${\hat{\pi }}_{k}$. This then ensures that Equation (30) is obeyed:

(46) $$\left[{\hat{\phi }}_{k},{\hat{\pi }}_{k^{\prime }}\right]=\frac{1}{2}\left[{\hat{\phi }}_{k}^{\left(1\right)}+i\;{\hat{\phi }}_{k}^{\left(2\right)},\;p_{k}^{\left(1\right)}-i\;p_{k}^{\left(2\right)}\right]=i\;\delta_{k,k^{\prime }}\;,$$

if $\left[{\hat{\phi }}_{k}^{\left(i\right)},\;p_{k^{\prime }}^{\left(j\right)}\right]=i\;\delta_{ij}\delta_{k,k^{\prime }}$. Furthermore, since ϕ^+k†=ϕ^−k†, we find:

(47) $$\begin{array}{cccccc} {\hat{\phi }}_{+k}^{\left(1\right)} & =+{\hat{\phi }}_{-k}^{\left(1\right)}\;, & \; & \mathrm{and}\;\;\; & {\hat{\phi }}_{+k}^{\left(2\right)} & =-{\hat{\phi }}_{-k}^{\left(2\right)}\;. \end{array}$$

That is, ${\hat{\phi }}_{k}^{\left(1\right)}$ is an even function of k, but ${\hat{\phi }}_{k}^{\left(2\right)}$ is an odd function of k. Here one may already expect that neither the operator ${\hat{\phi }}_{k}$ nor ${\hat{\phi }}_{k}^{\left(i\right)}$ can offer an unambiguous specification of the ±k modes.

To construct the density matrix operator, we need the covariance matrix elements of ${\hat{\phi }}_{k}^{\left(i\right)}$. This can be done by first inverting the decompositions (42) and (45)

(48) ϕ^k(1)=12ϕ^k†+ϕ^k†,    ϕ^k(2)=1i2ϕ^k†−ϕ^k†,

(49) p^k(1)=12π^k†+π^k†,    p^k(2)=1i2π^k†−ϕ^k†.

From (38)–(40), we obtain the covariance matrix elements of ${\hat{\phi }}_{k}^{\left(i\right)}$ with respect to the initial stationary state given by:

(50) 〈ϕ^k(1)2〉=〈N^+k〉+12+〈N^−k〉+12uω∗uω∗=〈ϕ^−k(1)2〉,

(51) 12〈ϕ^k(1),p^k(1)〉=12〈N^+k〉+12+〈N^−k〉+12uω∗u˙ω∗+u˙ω∗uω∗=12〈ϕ^−k(1),p^−k(1)〉,

and then

(52) $$\begin{array}{cccc} \langle {\hat{\phi }}_{k}^{(2)2}\rangle & =\langle {\hat{\phi }}_{k}^{(1)2}\rangle \;,\;\;\;\; & \frac{1}{2}\langle \left\{{\hat{\phi }}_{k}^{\left(2\right)},\;{\hat{p}}_{k}^{\left(2\right)}\right\}\rangle & =\frac{1}{2}\langle \left\{{\hat{\phi }}_{k}^{\left(1\right)},\;{\hat{p}}_{k}^{\left(1\right)}\right\}\rangle \;. \end{array}$$

Finally, we find:

(53) $$\begin{array}{cccc} \frac{1}{2}\langle \left\{{\hat{\phi }}_{k}^{\left(1\right)},\;{\hat{\phi }}_{k}^{\left(2\right)}\right\}\rangle & =0\;,\;\;\;\; & \frac{1}{2}\langle \left\{{\hat{\phi }}_{k}^{\left(1\right)},\;{\hat{p}}_{k}^{\left(2\right)}\right\}\rangle & =0\;, \end{array}$$

for the initial stationary state.

Having seen how the field modes are expressed in terms of harmonic oscillators, we can now construct the density matrix operators of the field modes.

### 5.1. Density Matrix Operator

Let us quickly review the general form of the density matrix operator of a Gaussian state [108]. Consider the one-mode case, and let $\left(\hat{q},\hat{p}\right)$ be the conjugated canonical operators of a Gaussian system. The density operator of the Gaussian state in general takes the form:

(54) $$\begin{array}{c} \hat{\rho }\left(\hat{q},\hat{p}\right)=\frac{2}{\sqrt{e^{2\chi }-1}}\;\exp\left\{-e^{-\chi }\cosh^{-1}\left(\coth\chi \right)\;\left[a\;{\hat{q}}^{2}+b\;{\hat{p}}^{2}-c\;\left\{\hat{q},\;\hat{p}\right\}\right]\right\}\;,\; \end{array}$$

where the coefficients can be expressed in terms of the corresponding time-dependent covariance matrix elements:

(55) $$\begin{array}{cccccccccc} a & =\langle {\hat{p}}^{2}\rangle \;,\; & b & =\langle {\hat{q}}^{2}\rangle \;,\; & c & =\frac{1}{2}\langle \left\{\hat{q},\hat{p}\right\}\rangle \;,\; & \; & \mathrm{and}\; & e^{2\chi } & =4\left(ab-c^{2}\right)\;. \end{array}$$

By means of the creation and annihilation operators, the density matrix operator (54) becomes:

(56) $$\begin{array}{c} \hat{\rho }\left(\hat{a},{\hat{a}}^{†}\right)=\frac{2}{\sqrt{e^{2\chi }-1}}\;\exp\left\{-2\Xi \;e^{-\chi }\cosh^{-1}\left(\coth\chi \right)\;\left[\kappa \;{\hat{a}}^{2}+\kappa^{*}\;{\hat{a}}^{†2}+\frac{\tau }{2}\;\left\{\hat{a},\;{\hat{a}}^{†}\right\}\right]\right\}\;,\; \end{array}$$

where

(57) $$\begin{array}{cccc} \kappa & =\frac{1}{2}\;\sinh2\eta \;e^{-i\psi }\;,\;\;\;\; & \tau & =\cosh2\eta \;,\; \end{array}$$

(58) $$\begin{array}{cccc} \Xi & =\coth\frac{\beta }{2}\;,\;\;\;\;\;\; & e^{2\chi } & =\Xi^{2}\left(\tau^{2}-4{\left|\kappa \right|}^{2}\right)=\Xi^{2}=\coth^{2}\frac{\beta }{2}\;,\; \end{array}$$

and β, $\zeta =\eta \;e^{i\psi }$ are inverse-temperature-like and squeeze-like parameters. Hence we have:

(59) $$\begin{array}{cccc} 2\Xi \;e^{-\chi }\cosh^{-1}\left(\coth\chi \right) & =2\beta \;,\; & \frac{2}{\sqrt{e^{2\chi }-1}} & =2\sinh\frac{\beta }{2}\;. \end{array}$$

The second expression in (59) is essentially the inverse of the nonequilibrium partition function [108] associated with the density matrix operator (54) or (56). Here, we have assumed that the first moments vanish for the state considered; otherwise, we may let $\hat{a}\to \hat{a}-\langle \hat{a}\rangle$ or $\hat{q}\to \hat{q}-\langle \hat{q}\rangle$ for the more general cases.

Observe that the factor:

(60) $$\kappa \;{\hat{a}}^{2}+\kappa^{*}\;{\hat{a}}^{†2}+\frac{\tau }{2}\;\left\{\hat{a},\;{\hat{a}}^{†}\right\}$$

can be ‘rotated’ into the form:

(61) $$\frac{1}{2}\;\left\{\hat{b},\;{\hat{b}}^{†}\right\}\;.$$

Suppose such a $\hat{b}$ is related to $\hat{a}$ and ${\hat{a}}^{†}$ by $\hat{b}=\mu \;\hat{a}+\nu \;{\hat{a}}^{†}$, with ${\left|\mu \right|}^{2}-{\left|\nu \right|}^{2}=1$, we have:

(62) $$\begin{array}{cc} \frac{1}{2}\;\left\{\hat{b},\;{\hat{b}}^{†}\right\} & =\frac{\mu \nu^{*}}{2}\;\left\{\hat{a},\hat{a}\right\}+\frac{\mu^{*}\nu }{2}\;\left\{{\hat{a}}^{†},{\hat{a}}^{†}\right\}+\frac{{\left|\mu \right|}^{2}+{\left|\nu \right|}^{2}}{2}\;\left\{\hat{a},{\hat{a}}^{†}\right\}\;. \end{array}$$

Comparing with (60), we obtain:

(63) $$\begin{array}{cccc} \mu \nu^{*} & =\kappa =\frac{1}{2}\;\sinh2\eta \;e^{-i\psi }\;,\;\;\; & {\left|\mu \right|}^{2}+{\left|\nu \right|}^{2} & =2\left({\left|\nu \right|}^{2}+\frac{1}{2}\right)=\tau =\cosh2\eta \;. \end{array}$$

If we assume μ∈R, then a useful choice of (μ,ν) is

(64) $$\begin{array}{cccc} \mu & =\cosh\eta \;,\;\;\;\;\;\;\; & \nu & =\sinh\eta \;e^{-i\psi }\;,\; \end{array}$$

that is, a squeeze transformation. A complex μ will correspond to an additional rotation, so that (64) will contain the phase factor that accounts for the rotation.

Since from (43), the real part and the imaginary parts of ${\hat{\phi }}_{k}$ behave like independent harmonic oscillators, we expect that the corresponding density operator for each k>0 mode will be:

(65) $$\begin{array}{cc} \hat{\rho }\left({\hat{\phi }}_{k}^{\left(1\right)},{\hat{\phi }}_{k}^{\left(2\right)}\right) & ={\left(\frac{2}{\sqrt{e^{2\chi_{k}}-1}}\right)}^{2}\;\exp\left\{-e^{-\chi_{k}}\cosh^{-1}\left(\coth\chi_{k}\right)\;\left[a_{k}^{\left(1\right)}{\hat{\phi }}_{k}^{(1)2}+b_{k}^{\left(1\right)}{\hat{p}}_{k}^{(1)2}\right.\right.\\ \; & \;-\left.\left.c_{k}^{\left(1\right)}\left\{{\hat{\phi }}_{k}^{\left(1\right)},\;{\hat{p}}_{k}^{\left(1\right)}\right\}+a_{k}^{\left(2\right)}{\hat{\phi }}_{k}^{(2)2}+b_{k}^{\left(2\right)}{\hat{p}}_{k}^{(2)2}-c_{k}^{\left(2\right)}\left\{{\hat{\phi }}_{k}^{\left(2\right)},\;{\hat{p}}_{k}^{\left(2\right)}\right\}\right]\right\}\;,\; \end{array}$$

where we have assumed that $\chi_{k}$ is the same for the real and the imaginary components, to be justified later. Equations (50)–(53) imply that:

(66) $$\begin{array}{cccccc} a_{+k}^{\left(2\right)} & =a_{+k}^{\left(1\right)}=a_{-k}^{\left(1\right)}=a_{-k}^{\left(2\right)}\;,\; & b_{+k}^{\left(2\right)} & =b_{+k}^{\left(1\right)}=b_{-k}^{\left(1\right)}=b_{-k}^{\left(2\right)}\;,\; & c_{+k}^{\left(2\right)} & =c_{+k}^{\left(1\right)}=c_{-k}^{\left(1\right)}=c_{-k}^{\left(2\right)}\;,\; \end{array}$$

so let us assign

(67) $$\begin{array}{cccccc} a_{k} & \equiv a_{k}^{\left(1\right)}=a_{k}^{\left(2\right)}\;,\;\;\;\; & b_{k} & \equiv b_{k}^{\left(1\right)}=b_{k}^{\left(2\right)}\;,\;\;\;\; & c_{k} & \equiv c_{k}^{\left(1\right)}=c_{k}^{\left(2\right)}\;,\; \end{array}$$

and write Equation (65) as:

(68) $$\begin{array}{cc} \hat{\rho }\left({\hat{\phi }}_{k}^{\left(1\right)},{\hat{\phi }}_{k}^{\left(2\right)}\right) & ={\left(\frac{2}{\sqrt{e^{2\chi_{k}}-1}}\right)}^{2}\\ \; & \;\times \exp\left\{-e^{-\chi_{k}}\cosh^{-1}\left(\coth\chi_{k}\right)\;\left[a_{k}\left({\hat{\phi }}_{k}^{(1)2}+{\hat{\phi }}_{k}^{(2)2}\right)+b_{k}\left({\hat{p}}_{k}^{(1)2}+{\hat{p}}_{k}^{(2)2}\right)\right.\right.\\ \; & \;\;\;\;\;\;\;-\left.\left.c_{k}\left(\left\{{\hat{\phi }}_{k}^{\left(1\right)},\;{\hat{p}}_{k}^{\left(1\right)}\right\}+\left\{{\hat{\phi }}_{k}^{\left(2\right)},\;{\hat{p}}_{k}^{\left(2\right)}\right\}\right)\right]\right\}\;,\; \end{array}$$

with k>0.

When we take into account all modes, the density matrix operator of the field modes $\hat{\rho }\left(\left\{{\hat{\phi }}_{k}\right\},\left\{{\hat{\pi }}_{k}\right\}\right)=\prod_{k>0}\hat{\rho }\left({\hat{\phi }}_{k}^{\left(1\right)},{\hat{\phi }}_{k}^{\left(2\right)}\right)$ corresponding to (43) can be found to be:

(69) ρ^({ϕ^k},{π^k})=∏k2e2χk−1exp−∑ke−χkcosh−1(cothχk)akϕ^+k†ϕ^+k†+bkπ^+k†π^+k†−ckϕ^k,π^k,

where we have assumed that the parameter $\chi_{+k}=\chi_{-k}$, to be justified later. What remains is to express the coefficients $b_{k}$, $a_{k}$ and $c_{k}$ by the original field mode operators ${\hat{\phi }}_{k}$ and ${\hat{\pi }}_{k}$. Since $b_{k}=\langle {\hat{\phi }}_{k}^{(1)2}\rangle =\langle {\hat{\phi }}_{k}^{(2)2}\rangle$, we find:

(70) bk=12〈ϕ^k(1)2〉+〈ϕ^k(2)2〉=12〈ϕ^k†ϕ^k†〉+〈ϕ^−k†ϕ^−k†〉=12〈ϕ^k†,ϕ^k†〉.

Likewise, from:

(71) $$\begin{array}{c} c_{k}=\frac{1}{2}\langle \left\{{\hat{\phi }}_{k}^{\left(1\right)},\;{\hat{p}}_{k}^{\left(1\right)}\right\}\rangle =\frac{1}{2}\langle \left\{{\hat{\phi }}_{k}^{\left(2\right)},\;{\hat{p}}_{k}^{\left(2\right)}\right\}\rangle \;,\; \end{array}$$

we obtain

(72) $$\begin{array}{c} c_{k}=\frac{1}{2}\left[\frac{1}{2}\langle \left\{{\hat{\phi }}_{k},\;{\hat{\pi }}_{k}\right\}\rangle +\frac{1}{2}\langle \left\{{\hat{\phi }}_{k}^{†},\;{\hat{\pi }}_{k}^{†}\right\}\rangle \right]=\frac{1}{2}\;\left[\frac{1}{2}\langle \left\{{\hat{\phi }}_{k},\;{\hat{\pi }}_{k}\right\}\rangle +\frac{1}{2}\langle \left\{{\hat{\phi }}_{-k},\;{\hat{\pi }}_{-k}\right\}\rangle \right]\;. \end{array}$$

At the first sight, the result for $c_{k}$ is not quite satisfactory because we cannot clearly separate contributions from the ±k modes. However, later we will see this is the form needed to construct the Wigner function of the whole field, instead of for each mode. This shows explicitly that there is an inseparable correlation between the ±k modes.

In terms of the mode expansion (31), the elements $b_{k}$, $a_{k}$ and $c_{k}$ take the forms:

(73) bk=〈N^+k〉+12+〈N^−k〉+12uω∗uω∗,

(74) ak=〈N^+k〉+12+〈N^−k〉+12u˙ω∗u˙ω∗,

(75) ck=12〈N^+k〉+12+〈N^−k〉+12uω∗u˙ω∗+u˙ω∗uω∗,

with N^k†=a^k†a^k†, if the initial state is stationary. We again observe that they are the same for ±k modes and thus the corresponding parameters $\chi_{k}$, $\beta_{k}$, $\zeta_{k}$, $\kappa_{k}$ and $\tau_{k}$ in (57) and (58) will also be the same for ±k modes, because they are functions of the covariance matrix elements $b_{k}$, $a_{k}$ and $c_{k}$. Note that from [108], we observe that the density matrix operator thus constructed is fairly general for the configuration under study. In addition, it is worth noticing that, since the second expression in (59) can be identified as the inverse of the nonequilibrium partition function for the canonical variables $\left({\hat{\phi }}_{k},{\hat{\pi }}_{k}\right)$, we find that the prefactor before the exponential in (69) turns out to be the inverse of the nonequilibrium partition function of the quantum field, from which we can extract the nonequilibrium free energy of the field. Since these quantities are nonequilibrium in nature, they are all functions of time. Moreover, since the field is driven by the parametric process, the state of the field at any moment is a general Gaussian state, given an initial Gaussian state. It means that the nonequilibrium partition function or the nonequilibrium free energy for such a Gaussian system is introduced in a context much more general than conventional equilibrium quantum thermodynamics.

### 5.2. Density Matrix Elements

The matrix elements of the density operator of a Gaussian state in the $\phi_{k}^{\left(i\right)}$ basis can be written as:

(76) ϱ(ϕk(i),ϕk′(i))=Nϱexp−Ak2Δk(i)2−i2Bk2Δk(i)Σk(i)−Ck2Σk(i)2,

where i=1, 2, and the relative coordinate $\Delta_{k}^{\left(i\right)}$, center-of-mass coordinate $\Sigma_{k}^{\left(i\right)}$, and normalization constant $N_{\varrho }$ are respectively given by:

(77) $$\begin{array}{cccccc} \Delta_{k}^{\left(i\right)} & =\phi_{k}^{\left(i\right)}-\phi_{k}^{\prime (i)}\;,\; & \Sigma_{k}^{\left(i\right)} & =\frac{\phi_{k}^{\left(i\right)}+\phi_{k}^{\prime (i)}}{2}\;,\; & N_{\varrho } & =\frac{\sqrt{C_{k}}}{\sqrt{\pi }}\;,\; \end{array}$$

and

(78) Ak=ak2bk2−ck22bk,Bk=−ck2bk,Ck=12bk,

with

(79) $$\begin{array}{cccccc} b_{k} & =\langle \phi_{k}^{(i)2}\rangle \;,\; & a_{k} & =\langle p_{k}^{(i)2}\rangle \;,\; & c_{k} & =\frac{1}{2}\langle \left\{\phi_{k}^{\left(i\right)},p_{k}^{\left(i\right)}\right\}\rangle \;. \end{array}$$

If the initial state of the density matrix is a vacuum state, then we always have:

(80) bk2ak2−ck2=14,

so that (76) reduces to:

(81) $$\begin{array}{cc} \varrho (\phi_{k}^{\left(i\right)},\phi_{k}^{\prime (i)}) & =N_{\varrho }\exp\left[-\frac{1}{4b_{k}}\left(\phi_{k}^{(i)2}+\phi_{k}^{\prime (i)2}\right)+i\;\frac{c_{k}}{2b_{k}}\left(\phi_{k}^{(i)2}-\phi_{k}^{\prime (i)2}\right)\right]\;. \end{array}$$

## 6. Evolution of Mode Functions: Bogoliubov Transformation

The dynamics of the field is fully encoded in its density matrix operator or its elements. The time evolution of the density matrix can be described by the unitary time-evolution operator $\hat{U}\left(t_{f},t_{i}\right)$ associated with the Lagrangian (24),

(82) $$\hat{\rho }\left(t_{f}\right)=\hat{U}\left(t_{f},t_{i}\right)\;\hat{\rho }\left(t_{i}\right)\;{\hat{U}}^{†}\left(t_{f},t_{i}\right)\;.$$

A more succinct description of the evolution of a linear quantum system is by using the language of Bogoliubov transformations in the Heisenberg picture. We begin with a brief overview of the Bogoliubov transformation.

### 6.1. Active and Passive Views of the Bogoliubov Transformation

Suppose the quantum scalar field $\hat{\phi }$ is expanded with respect to two different sets of mode functions $u_{k}^{\left(\mathrm{IN}\right)}\left(t\right)$ and $u_{k}^{\left(\mathrm{OUT}\right)}\left(t\right)$. Then according to (31) the expansion takes the form:

(83) ϕ^(x,t)=∑ka^k†uk(IN)(x,t)+a^k†uk(IN)∗(x,t),

or, in terms of the out-mode functions, in the form

(84) ϕ^(x,t)=∑kb^k†uk(OUT)(x,t)+b^k†uk(OUT)∗(x,t),

where ${\hat{a}}_{k}$ and ${\hat{b}}_{k}$ are the annihilation operators associated $u_{k}^{\left(\mathrm{IN}\right)}\left(t\right)$ and $u_{k}^{\left(\mathrm{OUT}\right)}\left(t\right)$ respectively. If both sets of mode functions are complete, then they will be related by a linear transformation:

(85) uk(IN)(x,t)=αk(OUT)uk(OUT)(x,t)+βk(OUT)u−k(OUT)∗(x,t),with uk(IN)(x,t)=eik·xuω(IN)(t),

where $\alpha_{k}$, $\beta_{k}\in C$ are called the Bogoliubov coefficients [11,109,110], satisfying:

(86) $$\begin{array}{c} {\left|\alpha_{k}\right|}^{2}-{\left|\beta_{k}\right|}^{2}=1\;,\; \end{array}$$

for the configuration of our interest, and $u_{\omega }^{\left(\mathrm{IN}\right)}\left(t\right)$ is the correspondence of $u_{\omega }\left(t\right)$ in (31) for the IN modes. In fact, Equation (85) is equivalent to:

(87) uω(IN)(t)=αk(OUT)uω(OUT)(t)+βk(OUT)uω(OUT)∗(t),

after we factor out $e^{ik\cdot x}$. Thus, for an isotropic, spatially-flat space, the Bogoliubov coefficients depend only on |k| or ω.

The annihilation operator ${\hat{b}}_{k}$ associated with the OUT mode function $u_{k}^{\left(\mathrm{OUT}\right)}$ will be related to the operators (a^k†,a^k†) by:

(88) b^k†=αk†a^k†+β−k†∗a^−k†.

This implies that in terms of ${\hat{b}}_{k}$ we have nonzero particle number $N_{k}$ and quantum coherence $C_{k}$

(89) Nk(OUT)=〈b^+k†b^+k†〉=|βk|2,

(90) Ck(OUT)=〈b^+kb^−k〉=αk∗βk∗,

in the vacuum state associated with ${\hat{a}}_{k}$ if $\beta_{k}\neq 0$, while in comparison, in terms of ${\hat{a}}_{k}$, they are:

(91) Nk(IN)=〈a^+k†a^+k†〉=0,

(92) $$\begin{array}{cc} C_{k}^{\left(\mathrm{IN}\right)} & =\langle {\hat{a}}_{+k}{\hat{a}}_{-k}\rangle =0\;. \end{array}$$

The physical meaning of these results will be explained later.

We may choose $\alpha_{k}$ in (86) to be real if we factor out the additional rotation in the transformation (85). This implies that generically the Bogoliubov coefficients can be parametrized by

(93) $$\begin{array}{cccc} \alpha_{k} & =\cosh\eta_{k}\;,\;\;\;\;\; & \beta_{k} & =-\sinh\eta_{k}\;e^{-i\theta_{k}}\;,\; \end{array}$$

with the time- and mode-dependent parameters $\eta_{k}>0$ and $0\leq \theta_{k}<2\pi$. These expressions remind us the squeeze transformation of the creation and annihilation operators in quantum optics. Actually, we can write the linear transformation (88) as the two-mode squeezing of ${\hat{a}}_{k}$ by the two-mode squeeze operator:

(94) S^2†(ζk†)=expζk∗†a^k†a^−k†−ζk†a^k†a^−k†,

with the squeeze parameter $\zeta_{k}=\eta_{k}\;e^{i\theta_{k}}$ such that:

(95) b^k†=S^2†(ζk†)a^k†S^2†(ζk†)=coshηk†a^k†−e+iθksinhηk†a^−k†.

When it applies to the vacuum state, it gives a two-mode squeezed vacuum state:

(96) |ζk(2)〉=S^2†(ζk†)|0+k,0−k〉=1coshηk∑n=0∞−tanhηke+iθkn|n+k,n−k〉,

in terms of the in-particle number states, and the corresponding density matrix operator is given by:

(97) $$\begin{array}{c} {\hat{\rho }}_{k}=|\zeta_{k}^{\left(2\right)}\rangle \langle \zeta_{k}^{\left(2\right)}|\;,\; \end{array}$$

still a pure state, but in general the off-diagonal terms n≠m of the density matrix do not vanish for each mode k. Equation (96) explicitly shows that the resulting state contains particles in pairs, with opposite momenta. Note that Equation (94) implies that $\zeta_{k}=\zeta_{-k}$, so that we use +k instead of −k for the squeeze parameters in (93). The identification (96) is compatible with the fact that the most general Gaussian state described by the density matrix operator (54) is a squeezed thermal state.

Relating the Bogoliubov coefficients to the squeeze operator in (95) provides an active view of the Bogoliubov transformation. It allows a mapping of an operator in the Heisenberg picture at one time to its counterpart at another time, that is, under time evolution. Or, alternatively, in the Schrödinger picture it maps the time evolution of the state. This is in contrast to the implementation of the Bogoliubov coefficients in (85), where they are used to relate different bases (mode expansion) of the same operator. This is the passive view. Hereafter, we will adopt the active view to investigate the time evolution of the parametric field.

Now from this active view, we see that the parametric process in general will drive the field from its initial vacuum state to a squeezed vacuum state, creating particles in pairs. It is also interesting to note that these $N_{k}$ and $C_{k}$ account for three parameters, needed to fully specify a Gaussian state, as $a_{k}$, $b_{k}$, and $c_{k}$ do, and thus they contain complete information about the linear quantum parametric field in a Gaussian state via the numbers of particle it creates and the correlation between the produced particle pairs.

The particles, as seen by ${\hat{b}}_{k}$, are correlated. According to the active view, Equation (89) only accounts for spontaneous creation since we have assumed that the initial state is a vacuum. If the initial state is not a vacuum state, but with $\langle {\hat{N}}_{k}^{\left(\mathrm{IN}\right)}\rangle$ particles present we can show that:

(98) ∑k>0〈N^+k(OUT)〉+〈N^−k(OUT)〉=∑k2|βk†|2+12〈N^k(IN)〉+12−12,

where 〈⋯〉 are taken with respect to the initial state. Thus, in addition to spontaneous particle creation, there is also stimulated creation due to the presence of particles in the initial state.

So far we have given a discussion of the generic kinematical properties of particle creation in a time-dependent background and made identification with quantum squeezing by the language of Bogoliubov transformations. We now show their connection with dynamics by using a nonequilibrium quantum dynamics formulation.

### 6.2. Nonequilibrium Evolution of Modes in Time

In the Heisenberg picture, the time evolution of the parametric field is accounted for by the (field) operators, and for the linear field, this information is encoded in the mode function $u_{\omega }\left(t\right)$. In other words, the time evolution of an operator can be translated into the time evolution of the mode function that span the operator. As an initial-value problem, in general, the mode function $u_{\omega }\left(t\right)$ at any time can be expanded by its initial conditions at t=0 by:

(99) $$u_{\omega }\left(t\right)=d_{\omega }^{\left(1\right)}\left(t\right)\;u_{\omega }\left(0\right)+d_{\omega }^{\left(2\right)}\left(t\right)\;{\dot{u}}_{\omega }\left(0\right)\;,$$

where the overdot represents the time derivative. The two functions $d_{\omega }^{\left(1\right)}\left(t\right)$ and $d_{\omega }^{\left(2\right)}\left(t\right)$ are a special set of homogeneous solutions to the wave Equation (26), satisfying:

(100) $$\begin{array}{cccccccc} d_{\omega }^{\left(1\right)}\left(0\right) & =1\;,\; & {\dot{d}}_{\omega }^{\left(1\right)}\left(0\right) & =0\;,\; & d_{\omega }^{\left(2\right)}\left(0\right) & =0\;,\; & {\dot{d}}_{\omega }^{\left(2\right)}\left(0\right) & =1\;. \end{array}$$

This approach is convenient in the sense that, for a linear system, almost all physical observables can be packaged into these two functions plus some initial conditions, so it would be computationally efficient once we get hold of the functions $d_{\omega }^{\left(1\right)}\left(t\right)$ and $d_{\omega }^{\left(2\right)}\left(t\right)$.

For convenience, suppose that the in-mode functions have the form:

(101) $$\begin{array}{cccccc} u_{\omega }^{\left(\mathrm{IN}\right)}\left(t\right) & =\frac{1}{\sqrt{2\omega_{i}}}\;e^{-i\omega_{i}t}\;,\; & \; & \mathrm{and}\;\; & {\dot{u}}_{\omega }^{\left(\mathrm{IN}\right)}\left(t\right) & =-i\sqrt{\frac{\omega_{i}}{2}}\;e^{-i\omega_{i}t}\;. \end{array}$$

In terms for the fundamental solutions $d_{\omega }^{\left(i\right)}$, we can write the out-mode functions as:

(102) $$u_{\omega }^{\left(\mathrm{OUT}\right)}\left(t\right)=\frac{1}{\sqrt{2\omega_{i}}}\;\left[d_{\omega }^{\left(1\right)}\left(t\right)-i\omega_{i}d_{\omega }^{\left(2\right)}\left(t\right)\right]\;,$$

according to (99), where $\omega_{i}=\omega \left(0\right)$ is the initial value of the frequency modulation. Thus, the field operator $\hat{\phi }\left(x,t\right)$ at time *t* will be given by:

(103) ϕ^(x,t)=∫d3k(2π)3212ωia^k†e+ik·xdω(1)(t)−iωidω(2)(t)+a^k†e−ik·xdω(1)(t)+iωidω(2)(t)

in contrast to the in-field before the parametric process starts at t=0.

(104) ϕ^IN(x,t)=∫d3k(2π)3212ωia^k†e+ik·xe−iωit+a^k†e−ik·xe+iωit.

When we apply the two-mode squeezing to this in-field, we can relate them by:

(105) ϕ^(x,t)=S^2†({ζk})ϕ^IN(x,t)S^2({ζk})=∫d3k(2π)3212ωiαk∗e−iωit+βk∗e+iωita^k†e+ik·x+βk∗e−iωit+αk∗e+iωita^k†e−ik·x,

where ${\hat{S}}_{2}\left(\left\{\zeta_{k}\right\}\right)$ is understood as a collection of two-mode squeeze operators for all k>0. Compare this expression with (103) and we find:

(106) uω(OUT)(t)=αk∗(t)uω(IN)(t)+βk∗(t)uω(IN)∗(t),⇒dω(1)(t)−iωidω(2)(t)=e−iωitαk∗(t)+e+iωitβk∗(t),

for t>0. Applying similar arguments to the conjugate momentum $\hat{\pi }\left(x,t\right)$, we will obtain:

(107) u˙ω(OUT)(t)=αk∗(t)u˙ω(IN)(t)+βk∗(t)u˙ω(IN)∗(t),⇒d˙ω(1)(t)−iωid˙ω(2)(t)=−iωie−iωitαk(t)+iωie+iωitβk(t).

Solving (106) and (107) simultaneously we arrive at:

(108) $$\begin{array}{cc} \alpha_{k}\left(t\right) & =\frac{1}{2\omega_{i}}\;e^{+i\omega_{i}t}\;\left[\omega_{i}\;d_{\omega }^{\left(1\right)}\left(t\right)+i\;{\dot{d}}_{\omega }^{\left(1\right)}\left(t\right)-i\;\omega_{i}^{2}d_{\omega }^{\left(2\right)}\left(t\right)+\omega_{i}\;{\dot{d}}_{\omega }^{\left(2\right)}\left(t\right)\right]\;,\; \end{array}$$

(109) $$\begin{array}{cc} \beta_{k}\left(t\right) & =\frac{1}{2\omega_{i}}\;e^{-i\omega_{i}t}\;\left[\omega_{i}\;d_{\omega }^{\left(1\right)}\left(t\right)-i\;{\dot{d}}_{\omega }^{\left(1\right)}\left(t\right)-i\;\omega_{i}^{2}d_{\omega }^{\left(2\right)}\left(t\right)-\omega_{i}\;{\dot{d}}_{\omega }^{\left(2\right)}\left(t\right)\right]\;. \end{array}$$

Thus we have expressed the Bogoliubov coefficients of all modes at any time by the corresponding fundamental solutions $d_{\omega }^{\left(i\right)}\left(t\right)$. Then from (93), we can write the squeeze parameters by the same set of fundamental solutions. For example, we can show:

(110) $${\left|\alpha_{k}\right|}^{2}+{\left|\beta_{k}\right|}^{2}=\frac{1}{2\omega_{i}^{2}}\left[\omega_{i}^{2}d_{\omega }^{(1)2}\left(t\right)+{\dot{d}}_{\omega }^{(1)2}\left(t\right)+\omega_{i}^{4}d_{\omega }^{(2)2}\left(t\right)+\omega_{i}^{2}{\dot{d}}_{\omega }^{(2)2}\left(t\right)\right]=\cosh2\eta_{k}\;.$$

This also implies that the squeeze parameters $\eta_{k}$, $\theta_{k}$ are actually functions of |k|.

The covariance matrix elements $b_{k}$, $a_{k}$ and $c_{k}$ can also be expressed by these fundamental solutions via the Bogoliubov coefficients. For example, from (48) and (31), we have:

(111) ϕ^k(1)(t)=12a^+k†uω∗(OUT(t)+a^−k†uω(OUT)∗(t)+a^−k†uω(OUT)∗(t)+a^+k†uω(OUT)∗(t),

and thus at any time:

(112) bk=〈ϕ^k(1)2〉=2|βk|2+12uω(IN)uω(IN)∗+αk∗βk∗uω(IN)2+αk∗βk∗uω(IN)∗2,

(113) ak=〈pk(1)2〉=2|βk|2+12u˙ω(IN)u˙ω(IN)∗+αk∗βk∗u˙ω(IN)2+αk∗βk∗u˙ω(IN)∗2,

(114) ck=12〈ϕk(1),pk(1)〉=|βk|2+12ddtuω(IN)uω(IN)∗+12αk∗βk∗ddtuω(IN)2+12αk∗βk∗ddtuω(IN)∗2.

Then we can further use (108) and (109) or simply use only (102) and (107). If the in-mode function is of the form (101), we can easily relate the covariance matrix elements with the particle number (89) and coherence (90) in the out-state by:

(115) $$\begin{array}{cc} b_{k}\left(t\right) & =d_{\omega }^{(1)2}\left(t\right)\;\frac{1}{2\omega_{i}}+d_{\omega }^{(2)2}\left(t\right)\;\frac{\omega_{i}}{2} \end{array}$$

(116) =Nk(OUT)(t)+121ωi+12ωiCk(OUT)∗(t)e−i2ωit+12ωiCk(OUT)∗(t)e+i2ωit,

(117) $$\begin{array}{cc} a_{k}\left(t\right) & ={\dot{d}}_{\omega }^{(1)2}\left(t\right)\;\frac{1}{2\omega_{i}}+{\dot{d}}_{\omega }^{(2)2}\left(t\right)\;\frac{\omega_{i}}{2} \end{array}$$

(118) =Nk(OUT)(t)+12ωi−ωi2Ck(OUT)∗(t)e−i2ωit−ωi2Ck(OUT)∗(t)e+i2ωit,

(119) $$\begin{array}{cc} c_{k}\left(t\right) & =d_{k}^{\left(1\right)}\left(t\right){\dot{d}}_{\omega }^{\left(1\right)}\left(t\right)\;\frac{1}{2\omega_{i}}+d_{\omega }^{\left(2\right)}\left(t\right){\dot{d}}_{k}^{\left(2\right)}\left(t\right)\;\frac{\omega_{i}}{2} \end{array}$$

(120) =−i2Ck(OUT)∗(t)e−i2ωit+i2Ck(OUT)∗(t)e+i2ωit,

or vice versa, write the particle number and coherence in terms of the fundamental solutions, attaining in principle all detailed information of their time evolution.

(121) $$\begin{array}{cc} N_{k}^{\left(\mathrm{OUT}\right)}\left(t\right) & =\sinh^{2}\eta_{k}\left(t\right)=\frac{1}{4}\left\{{\left[d_{\omega }^{\left(1\right)}\left(t\right)-{\dot{d}}_{\omega }^{\left(2\right)}\left(t\right)\right]}^{2}+{\left[\omega_{i}d_{\omega }^{\left(2\right)}\left(t\right)+\frac{1}{\omega_{i}}\;{\dot{d}}_{\omega }^{\left(1\right)}\left(t\right)\right]}^{2}\right\}\\ \; & =\frac{1}{4}\left[d_{\omega }^{(1)2}\left(t\right)+\frac{1}{\omega_{i}^{2}}\;{\dot{d}}_{\omega }^{(1)2}\left(t\right)+\omega_{i}^{2}d_{\omega }^{(2)2}\left(t\right)+{\dot{d}}_{\omega }^{(2)2}\left(t\right)\right]-\frac{1}{2}\;,\; \end{array}$$

and

(122) $$\begin{array}{cc} C_{k}^{\left(\mathrm{OUT}\right)}\left(t\right) & =-\frac{1}{2}\;\sinh2\eta_{k}\left(t\right)\;e^{+i\theta_{k}\left(t\right)}\\ \; & =\frac{e^{i2\omega_{i}t}}{4}\left\{{\left[d_{k}^{\left(1\right)}\left(t\right)+\frac{i}{\omega_{i}}\;{\dot{d}}_{k}^{\left(1\right)}\left(t\right)\right]}^{2}+\omega_{i}^{2}{\left[d_{k}^{\left(2\right)}\left(t\right)+\frac{i}{\omega_{i}}\;{\dot{d}}_{k}^{\left(2\right)}\left(t\right)\right]}^{2}\right\}\;. \end{array}$$

**Figure 1:**
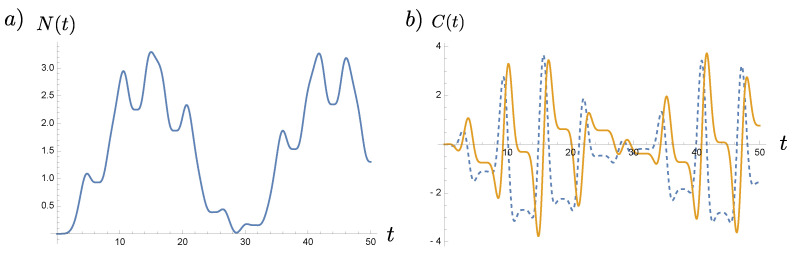
The time variations of the particle number N(t) in (**a**), and coherence C(t) in (**b**) for the parametric oscillator having the frequency modulation given by (123). We choose R=1 and ϖ=0.5. In (**b**) the blue dashed curve represents the real part of C(t) and the orange solid curve denotes the imaginary part.

**Figure 2:**
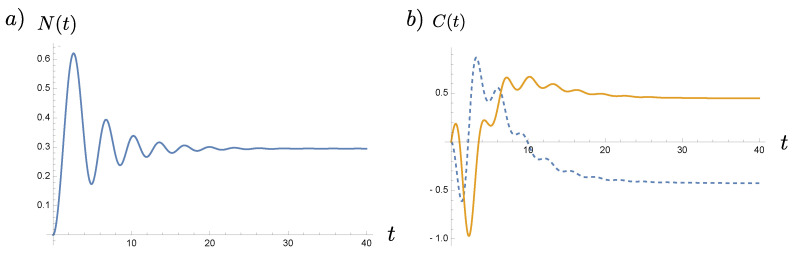
The time variations of the particle number N(t) in (**a**) and coherence C(t) in (**b**) for the parametric oscillator having the frequency modulation given by (124). We choose R=1 and ϖ=0.1. In (**b**) the blue dashed curve represents the real part of C(t) and the orange solid curve denotes the imaginary part.

We observe that, comparing with $N_{k}^{\left(\mathrm{OUT}\right)}\left(t\right)$, the coherence $C_{k}^{\left(\mathrm{OUT}\right)}\left(t\right)$ tends to oscillate more rapidly with time (but not necessarily always so), even though in principle both particle number and coherence are oscillatory, because $d_{\omega }^{\left(i\right)}$ are solutions of the parametric oscillator. To explicitly illustrate this point, we provide a few examples. First consider a parametric oscillator with the time-dependent frequency:

(123) $$\omega^{2}\left(t\right)=R^{2}\cos^{2}\varpi t\;,$$

where ϖ is the frequency of modulation and R is the amplitude. From Figure 1, we find that both N(t) and C(t) are oscillatory more or less at the same tempo except that N(t) is always nonnegative. Here we will suppress the superscript (OUT) for the moment because we are not comparing the results at two special instances of time. A contrasting example is the case with frequency.

(124) $$\omega^{2}\left(t\right)=R^{2}\left(1-e^{-2\varpi t}\right)\;,$$

where the frequency modulation monotonically rises from zero to a constant R. In this case, both N(t) and C(t) barely oscillate and quickly settle down to constant values, as shown in Figure 2. However, in the case of cosmological perturbation, if we consider (17) in the leading order and ignore the slow-roll parameter for the moment, we find that $N_{k}^{\left(\mathrm{OUT}\right)}$ happens to be non-oscillating and monotonically increases like $4^{-1}{\left(k\eta \right)}^{-4}$ as the conformal time $\eta \to 0^{-}$, but the coherence still oscillates with an increasing amplitude over the evolution toward the end of inflation, and the rate of oscillations depends on the mode of perturbations. The oscillatory behavior of the coherence will come to stop at about kη≃1, that is, the horizon crossing time, after which it evolves monotonically:

(125) $$\lim_{k\eta \ll 1}C_{k}\left(\eta \right)≃-\frac{1}{4k^{4}\eta^{4}}+i\;\frac{1}{k^{3}\eta^{3}}\;.$$

**Figure 3:**
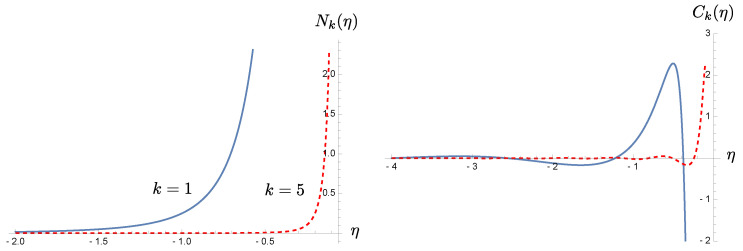
The time variations of the particle number $N_{k}\left(t\right)$ in (**a**) and coherence $C_{k}\left(t\right)$ in (**b**) for the Mukhanov-Sasaki variable *u* in the mode k in de Sitter spacetime. Here, the contribution of the slow-roll parameters in (17) is ignored. The particle number $N_{k}\left(t\right)$ monotonically increase with the time, but the coherence (here we only show the real part of $C_{k}\left(t\right)$) oscillates with a larger amplitude at a rate depending on the mode, until the perturbations cross the horizon at time $\eta ∼\frac{1}{k}$, after which it becomes non-oscillating. We choose k=1 for the blue solid curve and k=5 for the orange dashed curve.

Thus, for the superhorizon modes, both the particle number density and the coherence are non-oscillating. These can be seen in Figure 3. These examples illustrate our point that the contributions of quantum coherence need be taken more seriously, and arguments to justify its cancellation attributed to the rapid oscillations of coherence in comparison to particle number may be too simplistic. This observation is essential in the sense that our subsequent discussion on (possible) entropy production associated with particle creation applies to each mode of the field, and it does not involve the summation over a certain band of the field spectrum nor a measurement over a finite time duration.

For a Gaussian system, the physical quantities we are interested in can be built from the covariance matrix elements. We shall now focus on the von Neumann entropy.

## 7. Von Neumann Entropy of the Parametric Quantum Field

The von Neumann entropy $S_{k}$ for $\left(\phi_{k}^{\left(1\right)},p_{k}^{\left(1\right)}\right)$

(126) $$S_{k}=-\mathrm{Tr}\left\{\hat{\rho }\left({\hat{\phi }}_{k}^{\left(1\right)},{\hat{\pi }}_{k}^{\left(1\right)}\right)\;\ln\hat{\rho }\left({\hat{\phi }}_{k}^{\left(1\right)},{\hat{\pi }}_{k}^{\left(1\right)}\right)\right\}$$

can be given by the symplectic eigenvalue of the covariance matrix σ associated with the canonical variables $\left(\phi_{k}^{\left(1\right)},p_{k}^{\left(1\right)}\right)$

(127) $$S_{k}=(\lambda_{k}+\frac{1}{2})\;\ln(\lambda_{k}+\frac{1}{2})-(\lambda_{k}-\frac{1}{2})\;\ln(\lambda_{k}-\frac{1}{2})\;,$$

where the symplectic eigenvalue $\lambda_{k}$ is defined by:

(128) λk2=ak∗bk∗−ck2=〈ϕk(1)2〉〈pk(1)2〉−12〈{ϕk(1),pk(1)}〉2.

As a reminder, the symplectic eigenvalue λ of a 2N-dimensional covariance matrix σ can be found by solving the eigenvalue problem of the form

$$\sigma \cdot v=\lambda \;\Sigma \cdot v\;,\;\mathrm{or}\;\mathrm{equivalently}\;\left(\Sigma \cdot \sigma \right)\cdot v=\lambda \;v\;,$$

where the matrix Σ is the fundamental symplectic matrix

$$\Sigma =\overset{N}{\underset{k=1}{⨁}}\left(\begin{array}{cc} 0 & +i\\ -i & 0 \end{array}\right)\;.$$

From the covariance matrix elements (112)–(114), if the in-mode functions satisfy the Wronskian conditions, then we find the symplectic eigenvalues can be expressed by $N_{k}$ and $C_{k}$ as well,

(129) λk2=ak∗bk∗−ck2=−Nk(OUT)+122−|Ck(OUT)|2uk(IN)u˙k(IN)∗−u˙k(IN)uk(IN)∗2=(Nk(OUT)+12)2−|Ck(OUT)|2,

independent of the phase of $C_{k}^{\left(\mathrm{OUT}\right)}$ and independent of the explicit expressions of the in-mode functions. The latter merely reflects the invariance of the von Neumann entropy of the system during its unitary time evolution. The former implies that the phase of the coherence plays no role in evaluating the symplectic eigenvalue, and thus the von Neumann entropy. It further suggests we should not assume that a rapidly oscillating coherence can be canceled out and use this to smear off its contribution to the von Neumann entropy for each field mode.

Now we see that the entropy thus generated is formally somewhat different from that of a thermal state of the harmonic oscillator, or of a photon mode, we often come across:

(130) $$\begin{array}{cccc} S_{k} & =\left(N_{k}+1\right)\;\ln\left(N_{k}+1\right)-N_{k}\;\ln N_{k}\;,\;\;\; & N_{k} & =\frac{1}{e^{\beta \omega_{i}}-1}\;, \end{array}$$

by an additional coherence ${\left|C_{k}\right|}^{2}$. The expressions of $N_{k}$ is different too. At first sight, it is rather baffling why there exists any entropy (127) for a parametric process from a vacuum state, since the final state of each mode is a pure two-mode squeezed state. It is irrelevant to the basis we use in calculating the trace in (126) because the entropy is defined in terms of the trace and the eigenvalues $\lambda_{k}$ are invariant under the linear symplectic transformations. It is of importance to reconcile the paradoxical coexistence of the time-reversal of unitary evolution and the time irreversible entropy production.

The entropy (127), together with (128), seems to have a nonzero value, but the squeeze transformation alone does not change the value of the uncertainty function, that is, $\lambda_{k}^{2}$, so if the initial state before the parametric process is a vacuum state, whose entropy vanishes, then the resulting squeezed vacuum state due to the parametric process will also have zero entropy. This is consistent with the fact that both the vacuum state and the squeezed vacuum state are pure states, and thus their entropies are zero. Hence we expect (127) should vanish. Alternatively, since the density matrix of a closed system is governed by the Liouville equation:

(131) $$\frac{\partial \hat{\rho }\left(t\right)}{\partial t}=-i\;\left[\hat{H}\left(t\right),\hat{\rho }\left(t\right)\right]\;,$$

we can explicitly show that the von Neumann entropy is a constant of motion:

(132) $$\begin{array}{c} \frac{d}{dt}S\left(t\right)=-\frac{d}{dt}\mathrm{Tr}\hat{\rho }\left(t\right)\ln\hat{\rho }\left(t\right)=i\;\mathrm{Tr}\left\{\left[\hat{H}\left(t\right),\hat{\rho }\left(t\right)\right]\;\ln\hat{\rho }\left(t\right)\right\}=0\;, \end{array}$$

where we have used the cyclic property of the trace and

(133) $$\begin{array}{cccccc} \mathrm{Tr}\hat{\rho }\left(t\right) & =1\;, & \; & \Rightarrow & \mathrm{Tr}\frac{\partial \hat{\rho }\left(t\right)}{\partial t} & =0\;. \end{array}$$

That is, the entropy is indeed conserved under the unitary evolution. It implies that even the number of particles is changing, the entropy S remains the same.

On the other hand from (121) and (122) we can explicitly verify that indeed (129) is:

(134) $$\lambda_{k}^{2}={\left(N_{k}^{\left(\mathrm{OUT}\right)}+\frac{1}{2}\right)}^{2}-{\left|C_{k}^{\left(\mathrm{OUT}\right)}\right|}^{2}=\frac{1}{4}{\left[d_{k}^{\left(1\right)}\left(t\right){\dot{d}}_{k}^{\left(2\right)}\left(t\right)-{\dot{d}}_{k}^{\left(1\right)}\left(t\right)d_{k}^{\left(2\right)}\left(t\right)\right]}^{2}=\frac{1}{4}\;,$$

so that the corresponding entropy (127) of each field mode due to the parametric process does give zero, consistent with the earlier arguments. Thus we definitely will not have any entropy production for the parametric process evolving from the initial vacuum state.

At this point we have assumed that the field evolve unitarily, so the field evolves from a pure state into another one. We also assume that we can have full access to every bit of information the field possesses. However, in reality, a physical system is never perfectly closed, but coupled to some environment. When some information of the system is lost to its environment, or if some coarse-graining measure is introduced in the measurement protocols, entropy is observed to be generated in the reduced system. When a system undergoes nonequilibrium evolution some information is likely to be lost to the environment possessing a much larger number of degrees of freedom. The key point is coarse-graining, resulting in certain information becoming irretrievable. If we coarse-grain the information in the field we shall see nonzero entropy generated in the evolution of a pure state.

## 8. Evolution of the Wigner Function of the Quantum Field

In the context of creation of particle pairs, the Wigner function of the field offers the clearest picture of how the coarse-grained entropy may emerge, and a phase-space description offers an intimate view of its connection with quantum thermodynamics.

### 8.1. Wigner Function of the Field Mode

The Wigner function can be related to the density matrix elements (76) by a Fourier transformation,

(135) $$W_{k}^{\left(i\right)}\left(\Sigma_{k}^{\left(i\right)},P_{k}^{\left(i\right)};t\right)=\int \frac{du}{2\pi }\;e^{-iP_{k}^{\left(i\right)}\Delta_{k}^{\left(i\right)}}\;\rho \left(\Sigma_{k}^{\left(i\right)}+\frac{\Delta_{k}^{\left(i\right)}}{2},\Sigma_{k}^{\left(i\right)}-\frac{\Delta_{k}^{\left(i\right)}}{2};t\right)\;,$$

and we obtain:

(136) Wk(i)(Σk(i),Pk(i);t)=12πak2bk2−ck2exp−bk2Pk(i)2−2ck2Pk(i)Σk(i)+ak2Σk(i)22(ak2bk2−ck2).

The parameters $N_{k}$ and $C_{k}$ include the information we need about the Gaussian state of the linear parametric field. If we make the substitutions:

(137) bk=2Nk(OUT)+12uω(IN)uω(IN)∗+Ck(OUT)∗uω(IN)2+Ck(OUT)∗uω(IN)∗2,

(138) ak=2Nk(OUT)+12u˙ω(IN)u˙ω(IN)∗+Ck(OUT)∗u˙ω(IN)2+Ck(OUT)∗u˙ω(IN)∗2,

(139) ck=Nk(OUT)+12u˙ω(IN)uω(IN)∗+uω(IN)u˙ω(IN)∗+Ck(OUT)∗uω(IN)u˙ω(IN)+Ck(OUT)∗uω(IN)∗u˙ω(IN)∗,

and use the facts:

(140) uω(IN)∗(t)u˙ω(IN)∗(t)−uω(IN)∗(t)u˙ω(IN)∗(t)=i,Nk(OUT)+122−|Ck(OUT)|2=14

for the current setting, we find

(141) Wk(i)(Σk(i),Pk(i);t)=1πexp−22Nk(OUT)+12Dk(i)Dk(i)∗+Ck(OUT)∗Dk(i)2+Ck(OUT)∗Dk(i)∗2,

where $D_{k}^{\left(i\right)}=P_{k}^{\left(i\right)}u_{\omega }^{\left(\mathrm{IN}\right)}-\Sigma_{k}^{\left(i\right)}{\dot{u}}_{\omega }^{\left(\mathrm{IN}\right)}$. We have expressed the coefficients in the exponent of the Gaussian-state Wigner function in terms of $N_{k}$ and $C_{k}$.

Equation (141) is not quite what we want because it is written in terms of the real and the imaginary parts of the field modes. Rather we would like to write it as a function of the field modes $\phi_{k}$ and the conjugate momenta $\pi_{k}$. Inspired by (48) and (49)

(142) ϕ^k(1)=12ϕ^k†+ϕ^k†,ϕ^k(2)=1i2ϕ^k†−ϕ^k†,

(143) p^k(1)=12π^k†+π^k†,p^k(2)=1i2π^k†−ϕ^k†,

we will combine the Wigner functions $W_{k}^{\left(1\right)}\left(\Sigma_{k}^{\left(1\right)},P_{k}^{\left(1\right)};t\right)$ and $W_{k}^{\left(2\right)}\left(\Sigma_{k}^{\left(2\right)},P_{k}^{\left(2\right)};t\right)$ to form the Wigner function for the modes ±k:

(144) W±k(Σ+k,Π+k;Σ−k,Π−k;t)=Wk(1)(Σk(1),Pk(1);t)×Wk(2)(Σk(2),Pk(2);t)=1π2exp−22Nk(OUT)+12Dk(1)Dk(1)∗+Dk(2)Dk(2)∗+Ck(OUT)∗Dk(1)2+Dk(2)2+Ck(OUT)∗Dk(1)∗2+Dk(2)∗2.

Observe that we can write $D_{k}^{\left(1\right)}D_{k}^{(1)*}+D_{k}^{\left(2\right)}D_{k}^{(2)*}$ into

Dk(1)Dk(1)∗+Dk(2)Dk(2)∗=D+k∗D+k∗+D−k∗D−k∗,

in which we introduce a new set of variables $D_{\pm k}$,

(145) 12Dk(1)+iDk(2)=Π−kuω(IN)∗−Σ+ku˙ω(IN)∗≡D+k,

(146) 12Dk(1)−iDk(2)=Π+kuω(IN)∗−Σ−ku˙ω(IN)∗≡D−k,

with

(147) $$\begin{array}{cccc} \Pi_{k} & =\frac{1}{\sqrt{2}}\left(P_{k}^{\left(1\right)}-i\;P_{k}^{\left(2\right)}\right)\;,\; & \Sigma_{k} & =\frac{1}{\sqrt{2}}\left(\Sigma_{k}^{\left(1\right)}+i\;\Sigma_{k}^{\left(2\right)}\right)\;. \end{array}$$

Note that in general D+k∗≠D−k∗. Similarly we find:

(148) Dk(1)2+Dk(2)2=2D+k∗D−k∗,Dk(1)∗2+Dk(2)∗2=2D+k∗D−k∗.

Therefore Equation (144) becomes

(149) W±k(Σ+k,Π+k;Σ−k,Π−k;t)=1π2exp−4Nk(OUT)+12D+k∗D+k∗+D−k∗D−k∗+Ck(OUT)∗D+k∗D−k∗+Ck(OUT)∗D+k∗D−k∗.

We arrive at a Winger function in terms of a new set of variables $D_{\pm k}$, a superposition of the field mode $\Sigma_{k}$ and its conjugate momentum $\Pi_{k}$, but they have an unambiguous sense of parity due to D+k∗≠D−k∗, unlike $\Sigma_{k}$ and $\Pi_{k}$. In contrast, the latter set of variables in mode +k can be related to their counterparts in the −k mode by complex conjugation.

This nice feature allows us to unambiguously identify the effect of coarse-graining the field. Suppose we lose track of the information regarding the −k modes of the created particles, so we coarse-grain out the contribution of the −k mode in the Wigner Equation (149). Then we obtain:

(150) Wk(Σk,Πk;t)=∫dD−k∗dD−k∗W±k(Σ+k,Π+k;Σ−k,Π−k;t)=12π(Nk(OUT)+1/2)exp−1(Nk(OUT)+1/2)Dk∗Dk∗.

The resulting reduced Wigner function is independent of $C_{k}^{\left(\mathrm{OUT}\right)}$. It implies that the correlation between the particles of ±k is completely lost. Thus we expect the corresponding entropy will be a function of $N_{k}^{\left(\mathrm{OUT}\right)}$ only. In principle, a similar reduced-system argument can be carried out by taking the appropriate partial trace of the density matrix elements of the field (76). The eigenvalues of the resulting reduced density matrix elements can then be used to compute the associated von Neumann entropy. Both approaches will provide an equivalent description of the Gaussian system since the Wigner function and the density matrix elements are related by a Fourier transformation.

To better understand what (150) tells, and more specifically, the meaning of the variables $D_{k}$, we first note that (150) is essentially (141) with $C_{k}^{\left(\mathrm{OUT}\right)}=0$ and a rescaling of the variable $D_{k}^{\left(i\right)}$ by:

(151) $$D_{k}^{\left(i\right)}\mapsto \frac{1}{2N_{k}^{\left(\mathrm{OUT}\right)}+1}\;D_{k}^{\left(i\right)},$$

in (141), so we expect the corresponding entropy will be given by (127) with $C_{k}^{\left(\mathrm{OUT}\right)}=0$ in $\lambda_{k}$, that is,

(152) $$\begin{array}{cc} S_{k} & =\left(N_{k}^{\left(\mathrm{OUT}\right)}+1\right)\;\ln\left(N_{k}^{\left(\mathrm{OUT}\right)}+1\right)-N_{k}^{\left(\mathrm{OUT}\right)}\;\ln N_{k}^{\left(\mathrm{OUT}\right)}\;,\; \end{array}$$

with a corresponding particle number $N_{k}^{\left(\mathrm{OUT}\right)}$ for each k>0 mode. Thus the previous arguments lead to the conclusion that the field will have entropy production associated with the creation of particle pairs only when we lose track of some information embodied in the field—in this case, all the information about one partner of the pair. The nonzero entropy arises from the lack of complete information of the field, not from the creation of field quanta. Moreover, since the state of each field mode before coarse-graining is pure, the nonzero value of the von Neumann entropy (152) also indicates that the particle pair is entangled [74]. This may be alternatively seen if we decompose a two-mode squeezed state in terms of the particle number states of two modes as in (96). Though not obvious, the righthand side of (96) cannot be separated into a product of the states of ±k modes like $|\Psi_{+k}\rangle ⊗|\Phi_{-k}\rangle$. The von Neumann entropy provides an easy measure about this nonseparability. Thus now it is clear that what is deeper inside correlation or coherence between the particle pair is indeed quantum entanglement between them, as explicated in [74].

To find out the meaning of $D_{k}$, we first write the field by the out-mode functions,

(153) Σk=a+k∗uω(OUT)+a−k∗uω(OUT)∗,Πk=a−k∗u˙ω(OUT)+a+k∗u˙ω(OUT)∗,

where $a_{k}$ are the expansion coefficient, the *c*-number counterpart of the in-mode operator ${\hat{a}}_{k}$. Then, since the in-mode functions and the out-mode functions are related by:

(154) $$u_{\omega }^{\left(\mathrm{OUT}\right)}\left(t\right)=\alpha_{k}\;u_{\omega }^{\left(\mathrm{IN}\right)}+\beta_{k}\;u_{\omega }^{(\mathrm{IN})*}\;,$$

we find

(155) Dk=Πk∗uω(IN)∗−Σk∗u˙ω(IN)∗=αk∗a−k∗+βk∗a+k∗uω(IN)u˙ω(IN)∗−uω(IN)∗u˙ω(IN).

The second pair of parentheses give an *i* due to the Wronskian condition of the mode functions, while the expressions inside the first pair of parentheses is worth further investigation. From (88), we have:

(156) b−k∗=α−k∗a−k∗+β+k∗ak∗=αω∗a−k∗+βω∗ak∗,

because for our configuration the Bogoliubov coefficients are only functions of |k|. Thus we find that the variable Dk∗ actually is

(157) Dk∗=ib−k∗,

that is, expansion coefficient of $\Sigma_{k}$ with respect to the IN mode, so the Wigner function (150) now becomes

(158) Wk(t)=12π(Nk(OUT)+1/2)exp−1(Nk(OUT)+1/2)b−k∗b−k∗.

The labeling of the Wigner function can be a little awkward because now it is expanded by the variables of the −k mode.

It is interesting to note that, once we sever the correlation between the +k and the −k modes, the resulting Wigner function seems to be “instantaneously” stationary in the out-variables. However, it actually is not, because the associated coefficients are all time-dependent.

In summary, for the parametric processes of interest here, the field evolves unitarily, so even though particles are produced, there is no entropy production. Quantum entanglement exists between the created particle pairs. However, if some information of the field is lost resulting in some degree of nonunitarity, then the entropy of the field can increase even if the field is initially in a pure state. In particular, an entropy of the form (152) is obtained if information is lost associated with one of the particle pairs produced for each mode.

### 8.2. Wigner Function of the Quantum Field

It has been been shown that the statistical properties of the classical stochastic field can be described by a classical distribution functional of the field variable and its conjugate momentum over an infinite-dimensional phase space spanned by the canonical pair [1,2]. However, it is not explained in that context how a classical stochastic field can act as a full delegate of a quantum field. It will be more desirable to have a full quantum description for the statistical process of the field. Here, we provide such a formulation based on (136).

We will construct the Wigner function in terms of the full field ϕ(x,t), instead of its modes $\phi_{k}\left(t\right)$. From (136), we have the Wigner function of all modes given by:

(159) ∏k,iWk(i)(ϕk(i),pk(i);t)=∏k>0Nk(w)2exp−12∑k>0Ak2ϕk(1)2+ϕk(2)2+Bk2pk(1)2+pk(2)2+2Ck2ϕk(1)pk(1)+ϕk(2)pk(2),

where we note that $\phi_{k}^{\left(i\right)}$ and $p_{k}^{\left(i\right)}$ are *c*-number, and

(160) Nk(w)=12πak2bk2−ck2,Ak2=akak2bk2−ck2,

(161) Bk2=bkak2bk2−ck2,Ck2=−ckak2bk2−ck2.

Since following (48) and (49), we immediately have the *c*-number counterparts of (142) and (143) given by:

(162) ϕk(1)=12ϕk∗+ϕk∗,ϕk(2)=1i2ϕk∗−ϕk∗,

(163) pk(1)=12πk∗+πk∗,pk(2)=1i2πk∗−ϕk∗,

so that we obtain

(164) ϕk(1)2+ϕk(2)2=2ϕk∗ϕk∗=2ϕ−k∗ϕ−k∗=2ϕk∗ϕ−k∗=ϕk∗ϕk∗+ϕ−k∗ϕ−k∗,

(165) pk(1)2+pk(2)2=2πk∗πk∗,

(166) ϕk(1)pk(1)+ϕk(2)pk(2)=ϕk∗πk∗+ϕk∗πk∗=ϕk∗πk∗+ϕ−k∗π−k∗.

If the initial state is stationary, then from (66) and (67) we find:

(167) A+k2=A−k2,B+k2=B−k2,C+k2=C−k2.

and then we obtain

(168) ∏k,iWk(i)(ϕk(i),pk(i);t)=∏kNk(w)exp−12∑kAk2ϕk∗ϕk∗+Bk2πk∗πk∗+2Ck2ϕk∗πk∗.

Following some algebraic manipulations similar to that in [1,2], we observe that the functional “product” of the stationary functions,

(169) $$f\left(x-z\right)=\int_{-\infty }^{\infty }\;d^{3}y\;g\left(x-y\right)h\left(y-z\right),$$

can be written as

(170) $$\begin{array}{cc} f\left(x-z\right)=\int_{-\infty }^{\infty }\;d^{3}y\;g\left(x-y\right)h\left(y-z\right) & =\int \;\;\frac{d^{3}k}{{\left(2\pi \right)}^{3}}\;g_{k}h_{k}\;e^{+ik\cdot (x-z)}\;,\; \end{array}$$

and thus $f_{k}=g_{k}h_{k}$, where the spatial Fourier transformation is defined by

(171) $$f\left(x\right)=\int \;\;\frac{d^{3}k}{{\left(2\pi \right)}^{3}}\;f_{k}\;e^{+ik\cdot x}\;.$$

It implies that the inverse in this sense is given by

(172) $$\begin{array}{cccccccccc} \delta^{\left(3\right)}\left(x-z\right) & =\int_{-\infty }^{\infty }\;d^{3}y\;f\left(x-y\right)g\left(y-z\right)\;, & \; & \Rightarrow \;\; & 1 & =f_{k}g_{k}\;,\; & \; & \Rightarrow \;\; & f_{k}^{-1} & =\frac{1}{g_{k}}\;. \end{array}$$

That is, in this context, the notion of the inverse $g\left(x-y\right)=f^{-1}\left(x-y\right)$ of a function f(x−y) does not mean the usual inverse function, nor 1/f(x−y). Instead, their Fourier transforms obey the standard rule of the multiplication.

Therefore, the convolution relation for ϕ(x,t) reduces to

(173) ∫−∞∞d3x∫−∞∞d3yϕ(x,t)A(x−y;t)ϕ(y,t)=∫d3k(2π)3ϕk∗(t)Ak(t)ϕk∗(t),

On the other hand, the convolution relations are different for π(x,t) due to (28). For example we find

(174) ∫−∞∞d3x∫−∞∞d3yϕ(x,t)C(x−y;t)π(y,t)=∫d3k(2π)3ϕk∗(t)C−k∗(t)πk∗(t)=∫d3k(2π)3ϕk∗(t)Ck∗(t)πk∗(t),

and

(175) ∫−∞∞d3x∫−∞∞d3yπ(x,t)B(x−y;t)π(y,t)=∫d3k(2π)3πk∗(t)Bk∗(t)πk∗(t).

This implies that the Wigner function of the full field will be given by

(176) $$\begin{array}{cc} W(\phi ,\pi ;t) & =\prod_{k,i}W_{k}^{\left(i\right)}\left(\phi_{k}^{\left(i\right)},p_{k}^{\left(i\right)};t\right)\\ \; & =N^{\left(w\right)}\;\exp\left\{-\frac{1}{2}\int_{-\infty }^{\infty }\;d^{3}x\;\int_{-\infty }^{\infty }\;d^{3}y\left[\phi (x,t)A(x-y;t)\phi (y,t)\right.\right.\\ \; & \;\;\;\;+\left.\left.\pi (x,t)B(x-y;t)\pi (y,t)+2\phi (x,t)C(x-y;t)\pi (y,t)\right]\right\}\;,\; \end{array}$$

where we have used the fact that the covariance matrix elements

bk=12〈ϕ^k†,ϕ^k†〉,ak=12〈π^k†,π^k†〉,ck=1212〈ϕ^k,π^k〉+12〈ϕ^k†,π^k†〉,

are all real. The normalization constant $N^{\left(w\right)}$ will be determined by the functional integral,

(177) ∫DϕDπW(ϕ,π;t)=1.

The “inverse” of the coefficient functions A(x−y;t), B(x−y;t) and C(x−y;t) take simpler forms than the coefficient functions themselves because

(178) Ak−1=bk−ck−1ak−1ck−1,Bk−1=ak−ck−1bk−1ck−1,Ck−1=bk−ak−1ck−1bk−1.

Then, we find

A−1(x−y;t)=∫d3k(2π)3Ak−1e+ik·(x−y)=∫d3k(2π)3bk−ck1ak−1ck1e+ik·(x−y)

(179) $$\begin{array}{cc} \; & =b\left(x-y;t\right)-\int \;d^{3}ud^{3}v\;c\left(x-u;t\right)\;a^{-1}\left(u-v;t\right)\;c\left(v-y;t\right)\;,\; \end{array}$$

(180) $$\begin{array}{cc} B^{-1}\left(x-y;t\right) & =a\left(x-y;t\right)-\int \;d^{3}ud^{3}v\;c\left(x-u;t\right)\;b^{-1}\left(u-v;t\right)\;c\left(v-y;t\right)\;,\; \end{array}$$

(181) $$\begin{array}{cc} C^{-1}\left(x-y;t\right) & =c\left(x-y;t\right)-\int \;d^{3}ud^{3}v\;a\left(x-u;t\right)\;c^{-1}\left(u-v;t\right)\;b\left(v-y;t\right)\;,\; \end{array}$$

in the sense of functional multiplication. In deriving the above results we have used

(182) b(x−y;t)=∫d3k(2π)3bk(t)e+ik·(x−y)=∫d3k(2π)312〈ϕ^k†(t),ϕ^k†(t)〉e+ik·(x−y)=12〈ϕ^(x,t),ϕ^(y,t)〉,

due to (38), and similarly

$$a\left(x-y;t\right)=\int \;\;\frac{d^{3}k}{{\left(2\pi \right)}^{3}}\;a_{k}\left(t\right)\;e^{+ik\cdot (x-y)}=\frac{1}{2}\langle \left\{\hat{\pi }\left(x,t\right),\;\hat{\pi }\left(y,t\right)\right\}\rangle \;,$$

and

(183) $$\begin{array}{cc} c\left(x-y;t\right)=\;\int \;\;\frac{d^{3}k}{{\left(2\pi \right)}^{3}}\;c_{k}\left(t\right)\;e^{+ik\cdot (x-y)} & =\;\int \;\;\frac{d^{3}k}{{\left(2\pi \right)}^{3}}\frac{1}{2}\left[\frac{1}{2}\langle \left\{{\hat{\phi }}_{k},{\hat{\pi }}_{k}\right\}\rangle +\frac{1}{2}\langle \left\{{\hat{\phi }}_{k}^{†},{\hat{\pi }}_{k}^{†}\right\}\rangle \right]e^{+ik\cdot (x-y)}\\ \; & =\frac{1}{2}\;\langle \left\{\hat{\phi }\left(x,t\right),\;\hat{\pi }\left(y,t\right)\right\}\rangle \;,\; \end{array}$$

owing to (40). Again we remind that $A^{-1}\left(x-y;t\right)$ does not represent the reciprocal of A(x−y;t) per se.

We therefore see that the distribution function of the classical stochastic field is interpreted as the Wigner function (176) of the quantum scalar field. This may not come as a surprise. For the Gaussian system we consider here, the Wigner function is positive definite because the covariance matrix elements $a_{k}$ and $b_{k}$ are positive and they satisfy $a_{k}b_{k}-c_{k}^{2}>0$. Thus, it can serve as a classical probability distribution. Although this seems to justify the results obtained from classical stochastic field theory as in [1,2], the latter theory is not quite self-contained. It still needs inputs from the two-point functions of the quantum field to yield the stochastic counterparts. This bridging protocol is the source of persistent confusion in the literature so we to provide a pure quantum field theory approach. Thus, even though both look identical formally, the frameworks they are base upon are drastically distinct. Other than the lurid difference of quantum non-commutativity (In the classical stochastic field approach, different distribution functions may be needed to account for different operator orderings in quantum theory. This may be seen from the fact that in quantum theory the Wigner function corresponds to a *fully symmetrized ordering* [111,112,113]). Since in the current case, the quantities of interest can be expressed in terms of the covariance matrix elements, which are the expectation values of the canonical operators in symmetrized ordering, that may explain why the stochastic field approach may work, there are a few additional subtleties. The Wigner functions for the more general non-Gaussian configurations are not positive-definite, they carry more information than what is in the corresponding classical probability distributions. Classical field theory cannot account for quantum entanglement, in which the nature of quantum state plays an important role. Moreover, the Shannon entropy associated with classical distributions has the property of monotonicity. That is, given the combined systems *A* and *B*, we have $S_{s}\left[A+B\right]\geq \max\left\{S_{s}\left[A\right],S_{s}\left[B\right]\right\}$ for the Shannon entropy, where $S_{s}\left[A\right]$ denotes the Shannon entropy of the subsystem *A*. On the other hand, for the von Neumann entropy S, the closest property to the classical monotonicity is the theorem of Araki–Lieb triangle inequality:

(184) |S[A]−S[B]|≤S[A+B]≤S[A]+S[B].

The second inequalities is known as the subadditivity inequality for von Neumann entropy, and holds with equality if and only if systems *A* and *B* are uncorrelated, that is, $\varrho^{AB}=\varrho^{A}⊗\varrho^{B}$. In the case we discussed earlier, let the +k mode be the subsystem *A*, and the −k mode the subsystem *B*. Then we have shown S[A,B]=0 but S[A]=S[B]>0. The entropy of a subsystem is larger than the entropy of the combined system. These common properties are now often invoked in the discussions of entanglement entropy.

## 9. Discussion and Summary

In this concluding section we begin with a short description of how to apply what we have learned from the analysis of the nonequilibrium dynamics of squeezed quantum systems to the cosmological perturbation problem. We can only provide some useful pointers here, leaving a fuller implementation to subsequent work. A summary of this paper follows, addressing the objective, the methodology and our findings in some key issues.

### 9.1. Return to Cosmological Perturbations

A good portion of our effort in this work is devoted to the analysis of the evolution of a quantum field under a time-dependent parametric drive (squeezing) and how these results can be applied to the study of particle production and entropy generation as well as addressing the issues of quantum correlations, coherence and entanglement in flat space. At first sight it seems too remotely related to those of the cosmological perturbations in an inflationary universe. The connection between the parametric scalar field in flat space and the cosmological perturbations in (near) de Sitter space lies in (16), where, in terms of the Mukhanov–Sasaki variable, the cosmological perturbations follow an equation of motion of the exact form as for a parametric field in flat space. (Not even a conformal transformation is needed, and in flat space there is no distinction between conformal time and ordinary time). The gravitational effects of the cosmological spacetime is packaged in the function z(η), as can be seen in (16). Thus our results can be readily used for the Mukhanov–Sasaki variable. Since neither the scalar field perturbation δϕ and scalar component of the metric perturbation Φ is gauge or coordinate independent, the Mukhanov–Sasaki variable, a linear combination of them according to (15), provides a gauge-invariant description for the cosmological perturbations. This allows us to take a more direct route in applying the present results to the cosmological perturbation problem. We may choose a convenient gauge, such as the spatially-flat gauge [42,61], in which the scalar sector of the metric perturbations is completely turned off. In this gauge the Mukhanov–Sasaki variable for the cosmological perturbations becomes proportional to the field perturbation only, u(x,η)=a(η)δϕ(x,η). Thus all the results we have presented on entropy generation and quantum entanglement can be readily applied to both u(x,η) and δϕ(x,η), with the same physical contents in the context of cosmological perturbations. The minor difference only lies in the functional form of the frequency modulation.

### 9.2. Objective: Entropy of Quantum Fields and Cosmological Perturbations

We now provide a summary of the objective, our method and the issues explored.

A central subject of interest in cosmology is structure formation. The classical theory began with Lifshitz’s gravitational perturbation theory in 1946. Another important subject is particle creation from the quantum vacuum, believed to be abundant at the Planck time, first explored by Parker in 1966. Both aspects enter in theories of cosmological perturbations from inflationary cosmology, in 1982, where a quantum field is supposed to drive the universe into inflation and its fluctuations engender structures.

The entropy budget of the universe generated from various sources is also an essential concern in cosmology. Main focus was placed on particle processes at various stages of the cosmic evolution. Entropy associated with cosmological perturbations met with a new level of challenge after inflation took center stage. A necessary ingredient is to understand the entropy of quantum field processes, for free [4] and interacting fields [5]. That began in 1986. Incorporating both the quantum theory of gravitational perturbations [42] and the ideas about the entropy of quantum fields into a theory for the entropy of cosmological perturbations took shape in 1993 in the work of [1,2].

Our present work attempts to provide a synthesis of all the essential elements on this subject matter since that time, constructed from a more rigorous and comprehensive theoretical framework based on the nonequilibrium quantum field theory of squeezed quantum systems. We highlight its advantages as follows.

### 9.3. Methodology: Nonequilibrium Dynamics of Squeezed Quantum Systems

The technical core of this theory as applied to the present problem was presented in Section 5, Section 6 and Section 8 while the results meeting our objective, in Section 7. The systematic theoretical development of the formalism can be found in [26,108,109,114]. Applying this theory for squeezed open quantum systems, as long as they are Gaussian, enables us to obtain exact solutions for the fully nonequilibrium time-evolution of the cosmological perturbations, up to the Gaussian level.

We mention a few specific advantages: Essentially all quantities of interest can be expressed in terms of two fundamental solutions of the equation of motion of the system plus initial conditions. This make it computationally efficient, and easier to identify the structures of the models, compared with some earlier approaches, say [11]. With an exact formulation one can leave the necessary mathematical approximations or physical procedures, such as the regularization of the stress tensor associated produced particles’ density to the very end of the calculations where the actual expression is finally need.

A very useful tool special to Gaussian systems is the covariance matrix, whose power lies in the fact that the calculations involving the infinite-dimensional density matrix elements can be reduced to those of a handful of covariance matrix elements. The authors of [19] have used them to compute the von Neumann entropy of the field, but they can be also be used to form the symplectic invariants [115] and the observables of the Gaussian systems. These physical quantities are very useful for various applications in the context of open quantum systems like dynamical equilibration [116,117], quantum entanglement [118], nonequilibrium quantum thermodynamics [114,119].

In this work, we follow the same spirit. We first express the quantities of interest, not just limited to the von Neumann entropy, in terms of the covariance matrix elements. We then will express these elements in terms of the aforementioned fundamental solutions. One can break down a complex calculation to modular forms, which makes the comparisons of results from different theories easier to identify, and be able to go further as needed, sailing along guided by the formalism.

### 9.4. Issues: Quantum Correlations, Coherence, Phases

Entropy generation from quantum cosmological perturbations, as explained above, is weighted upon the issue of entropy of quantum fields associated with vacuum particle creation. Cosmological expansion having the same effect on a quantum state as squeezing, the issue becomes that of entropy generation from a squeezed quantum system. This is a good entry point to dissect the evolving explanations of entropy generation from quantum cosmological perturbations. We also see this as a good intellectual exercise which can reveal the subtleties of this issue, as it bears on the concepts of quantum correlations, coherence and what (not) to do with the phase.

Intuitively one may easily attribute entropy generation to the particle number from pair creation, as a result of the parametric amplification of the vacuum. However, an actual calculation shows that, starting in a vacuum state, the field will end up in the two-mode squeezed vacuum stage after the parametric process, which is a pure state and no entropy change. The authors of [4] pointed out that if one chooses to describe the particle creation process in a Fock representation and totally ignores their phase relations one would come up with this conclusion. Entropy is ‘generated’ because one choose to ignore certain information of the field which could be just as important. How one deals with the phase information is a different story. A common argument is to appeal to the random phase approximation (RHA) which assumes that rapid phase oscillation tends to smear out its own footprints. However the relevant phases are rather illusive. Let us examine this issue more closely.

Phase information can be placed in two categories: (1) the phase acquired during the evolution residing in the changes of the rotation phase plus the squeeze phase read off from the Bogoliubov coefficients; (2) initial phase of the squeeze parameter or the state. The former is fixed and dynamically determined, but the latter is largely unknown and in principle cannot be canceled as such. Thus the information of the latter is mostly inaccessible. The authors of [85] concretized this by writing down the quantum Vlasov equations of the particle number density and the coherence of the created particles, in an attempt to establish the connection between quantum coherence and the particle creation process which is non-Markovian. They argued that a nonzero entropy production associated with particle creation can emerge, if the variables associated with rapid phases are either plainly discarded or averaged out. This strategy is also adopted in [1,2,3]. However, this way to resolve the issue is not completely satisfactory in the sense that: (1) both the particle number density and coherence may be oscillatory in time for the parametric process, so even though the coherence may oscillate more rapidly than the particle number does, the difference is not always dramatic enough to justify discarding the coherence; (2) The authors of [74] show an example that coherence does not always oscillate. In particular, in the inflation setting, the coherence actually becomes non-oscillatory after the modes of the cosmological perturbations crossed the Hubble horizon; and (3) the low momentum modes have slow oscillation to make cancellation less effective. In addition, resorting to phase cancellation over all modes does not apply here because the emergent entropy occurs in each mode.

There is no denying that coherence tends to cancel because it is not positive definite as is the particle number density. Nonetheless it seems more reasonable to go after the essence of the coherence rather than its phases literally. The authors of [74] further argued that behind the coherence between pair-produced particles, more precisely, there is quantum entanglement between them at play. They showed the clear presence of entanglement by the von Neumann entropy of the reduced system after the degree of freedom of one party in the created particle pair is completely traced out. One can treat this as the emergent entropy associated with particle creation once one completely ignores or lose track of the information of one party in the particle pair. This we view as a better explanation of the root cause of emergent entropy associated with particle creation, and by extension, with cosmological perturbations involving quantum fields like in the inflationary universe.

We regained the result in [74] using a totally different formulation, based on the nonequilibrium dynamics of squeezed quantum fields. A quantum description is necessary for addressing the foundational issues of quantum decoherence and quantum entanglement, and a nonequilibrium dynamics formulation is necessary for time-dependent situations such as in a cosmological evolution. In relation to this we showed that a classical stochastic description of the quantum field often used, such as in [1,2], where quantum observables are believed to be obtainable by taking suitable ensemble averages of a classical stochastic field, though attractively simple and works in some aspects (A classical stochastic theory can be used to calculate certain quantum features of a Gaussian system, but it can not describe them in full. For example, once the classical stochastic theory acquires the two-point functions from the quantum field inputs, it can be used to calculate the covariance matrix elements, from which the entanglement measure can be computed. However there is no way for a classical stochastic theory to formulate, say, an entangled state, because it cannot account for the amplitude. In the end one still needs inputs from the quantum theory), proves inadequate to capture important quantum attributes fundamental to quantum information issues. The difficulties of attempting to substitute a quantum field by classical stochastic fields become almost insurmountable when one confronts nonGaussian systems. There, the Wigner function, known to possess the full information contained in a density matrix, is non-positive definite and can no longer be interpreted as a probability distribution. That is where the more intricate quantum features of the system reside.

## Data Availability

Not applicable.

## References

[1] Brandenberger R.H., Mukhanov V.F., Prokopec T. (1992). Entropy of a classical stochastic field and cosmological perturbations. Phys. Rev. Lett..

[2] Brandenberger R.H., Prokopec T., Mukhanov V.F. (1993). The entropy of the gravitational field. Phys. Rev. D.

[3] Prokopec T. (1993). Entropy of the squeezed vacuum. Class. Quant. Grav..

[4] Hu B.L., Pavon D. (1986). Intrinsic measures of field entropy in cosmological particle creation. Phys. Lett. B.

[5] Hu B.L., Kandrup H.E. (1987). Entropy generation in cosmological particle creation and interactions: A statistical subdynamics analysis. Phys. Rev. D.

[6] Habib S., Kandrup H.E. (1989). Wigner functions and density matrices in curved spaces as computational tools. Ann. Phys. (N.Y.).

[7] Calzetta E., Hu B.L. (1988). Nonequilibrium Quantum Fields: Closed-time-path effective action, Wigner function and Boltzmann equation. Phys. Rev. D.

[8] Gasperini M., Giovannini M. (1993). Entropy production in the cosmological amplification of the vacuum fluctuations. Phys. Lett. B.

[9] Gasperini M., Giovannini M. (1993). Quantum squeezing and cosmological entropy production. Class. Quant. Grav..

[10] Gasperini M., Giovannini M. (1998). Von Neumann and Shannon-Wehrl entropy for squeezed states and cosmological particle production. String Theory in Curved Space Times.

[11] Albrecht A., Ferreira P., Joyce M., Prokopec T. (1994). Inflation and squeezed quantum states. Phys. Rev. D.

[12] Calzetta E.A., Hu B.L. (1995). Quantum fluctuations, decoherence of the mean field, and structure formation in the early universe. Phys. Rev. D.

[13] Calzetta E.A., Hu B.L. (1999). Stochastic dynamics of correlations in quantum field theory: From Schwinger-Dyson to Boltzmann-Langevin equation. Phys. Rev. D.

[14] Calzetta E.A., Hu B.L. (2003). Correlation entropy of an interacting quantum field and *H*-Theorem for the *O*(*N*) Model. Phys. Rev. D.

[15] Kiefer C., Polarski D., Starobinsky A.A. (2000). Entropy of gravitons produced in the early universe. Phys. Rev. D.

[16] Kiefer C., Lohmar I., Polarski D., Starobinsky A.A. (2007). Pointer states for primordial fluctuations in inflationary cosmology. Class. Quant. Grav..

[17] Anderson P.R., Molina-Paris C., Mottola E. (2005). Short distance and initial state effects in inflation: Stress tensor and decoherence. Phys. Rev. D.

[18] Koksma J.F., Prokopec T., Schmidt M.G. (2010). Entropy and correlators in quantum field theory. Ann. Phys. (Amsterdam).

[19] Campo D., Parentani R. (2008). Decoherence and entropy of primordial fluctuations. II. The entropy budget. Phys. Rev. D.

[20] Boyanovsky D. (2015). Effective Field theory during in inflation: Reduced density matrix and its quantum master equation. Phys. Rev. D.

[21] Burgess C.P., Holman R., Tasinato G., Williams M. (2015). EFT beyond the horizon: Stochastic inflation and how primordial quantum fluctuations go classical. JHEP.

[22] Brahma R., Alaryani O., Brandenberger R.H. (2020). Entanglement entropy of cosmological perturbations. Phys. Rev. D.

[23] Rammer J. (2009). Quantum Field Theory of Non-Equilibrium States.

[24] Kamenev A. (2011). Field Theory of Non-Equilibrium Systems.

[25] Weiss U. (2012). Quantum Dissipative Systems.

[26] Calzetta E.A., Hu B.L. (2008). Nonequilibrium Quantum Field Theory.

[27] Breuer H.P., Petruccione F. (2007). The Theory of Open Quantum Systems.

[28] Rivas A., Huelga S.F. (2012). Open Quantum Systems: An Introduction.

[29] de Vega I., Alonso D. (2017). Dynamics of non-Markovian open quantum systems. Rev. Mod. Phys..

[30] Lifshitz E.M. (1946). On the gravitational stability of the expanding universe. J. Phys. (USSR).

[31] Lifshitz E.M., Khalatnikov I.M. (1963). Investigations in relativistic cosmology. Adv. Phys..

[32] Hawking S.W. (1966). Perturbations of an expanding universe. Astrophys. J..

[33] Peebles P.J., Yu J.T. (1970). Primeval adiabatic perturbation in an expanding universe. Astrophys. J..

[34] Bardeen J.M. (1980). Gauge invariant cosmological perturbations. Phys. Rev. D.

[35] Linde A.D. (1990). Particle Physics and Inflationary Cosmology.

[36] Mukhanov V.F. (2005). Physical Foundations of Cosmology.

[37] Guth A.H. (1981). Inflationary universe: A possible solution to the horizon and flatness problems. Phys. Rev. D.

[38] Hawking S.W. (1982). The development of irregularities in a single bubble inflationary universe. Phys. Lett. B.

[39] Guth A.H., Pi S.Y. (1982). Fluctuations in the new inflationary universe. Phys. Rev. Lett..

[40] Starobinsky A.A. (1982). Dynamics of phase transition in the new inflationary universe scenario and generation of perturbations. Phys. Lett. B.

[41] Bardeen J.M., Steinhardt P.J., Turner M.S. (1983). Spontaneous creation of almost scale-free density perturbations in an inflationary universe. Phys. Rev. D.

[42] Mukhanov V.F., Feldman H.A., Brandenberger R.H. (1992). Theory of cosmological perturbations. Phys. Rep..

[43] Lyth D.H., Liddle A.R. (2009). The Primordial Density Perturbation: Cosmology, Inflation and the Origin of Structure.

[44] Birrell N.D., Davies P.C.W. (1982). Quantum Fields in Curved Space.

[45] Hu B.L., Verdaguer E. (2020). Semiclassical and Stochastic Gravity: Quantum Field Effects on Curved Spacetime.

[46] Fulling S.A. (1989). Aspects of Quantum Field Theory in Curved Spacetime.

[47] Wald R.M. (1994). Quantum Field Theory in Curved Spacetime and Black Hole Thermodynamics.

[48] Parker L., Toms D. (2009). Quantum Field Theory in Curved Spacetime: Quantized Fields and Gravity.

[49] Zel’dovich Y.B. (1970). Particle production in cosmology. Pis’ma Zh. Eksp. Teor. Fiz..

[50] Parker L. (1969). Quantized fields and particle creation in expanding universes. I. Phys. Rev..

[51] Milton K.A., Bordag M. (2009). Proceedings of the Ninth Conference on Quantum Field Theory under the Influence of External Conditions (QFEXT09).

[52] Walls D.F. (1983). Squeezed states of light. Nature.

[53] Loudon R., Knight L.P. (1987). Squeezed light. J. Mod. Opt..

[54] Mandel L., Wolf E. (1995). Optical Coherence and Quantum Optics.

[55] Drummond P.D., Ficek Z. (2013). Quantum Squeezing.

[56] Grishchuk L.P., Sidorov Y.V. (1990). Squeezed quantum states of relic gravitons and primordial density fluctuations. Phys. Rev. D.

[57] Hu B.L., Kang G., Matacz A. (1994). Squeezed vacua and the quantum statistics of cosmological particle creation. Int. J. Mod. Phys. A.

[58] Hu B.L., Matacz A. (1994). Quantum Brownian motion in a bath of parametric oscillators: A model for system-field interactions. Phys. Rev. D.

[59] Anastopoulos C., Hu B.L. (2014). Problems with the Newton-Schrödinger equations. N. J. Phys..

[60] Anastopoulos C., Hu B.L. (2020). Quantum superposition of two gravitational cat states. Class. Quant. Grav..

[61] Weinberg S. (2008). Cosmology.

[62] Hu B.L. (2009). Emergent/quantum gravity: Macro/micro structures of spacetime. J. Phys. Conf. Ser..

[63] Roura A., Verdaguer E. (2008). Cosmological perturbations from stochastic gravity. Phys. Rev. D.

[64] Guth A.H., Pi S.Y. (1985). Quantum mechanics of the scalar field in the new inflationary universe. Phys. Rev. D.

[65] Starobinsky A.A. (1986). Stochastic de Sitter (inflationary) stage in the early universe. Field Theory, Quantum Gravity and Strings.

[66] Winitzki S., Vilenkin A. (2000). Effective noise in a stochastic description of inflation. Phys. Rev. D.

[67] Brandenberger R.H., Laflamme R., Mijic M. (1990). Classical perturbations from decoherence of quantum fluctuations in the inflationary universe. Mod. Phys. Lett. A.

[68] Lombardo F.C., Mazzitelli F.D. (1996). Coarse graining and decoherence in quantum field theory. Phys. Rev. D.

[69] Lombardo F.C., Nacir D.L. (2005). Decoherence during inflation: The generation of classical inhomogeneities. Phys. Rev. D.

[70] Matacz A. (1997). A new theory of stochastic inflation. Phys. Rev. D.

[71] Kiefer C., Polarski D., Starobinsky A.A. (1998). Quantum-to-classical transition for fluctuations in the early universe. Int. J. Mod. Phys. D.

[72] Hu B.L., Paz J.P., Zhang Y., Gunzig E., Nardone P. (1993). Quantum origin of noise and fluctuations in cosmology. The Origin of Structure in the Universe, Proceedings of the International Conference, Chateau de Pont d’Oye, Belgium, 27 April 1992.

[73] Hsiang J.-T., Hu B.L. No intrinsic decoherence of cosmological perturbations associated with a non-interacting quantum field. Universe.

[74] Lin S.-Y., Chou C.-H., Hu B.L. (2010). Quantum entanglement and entropy in particle creation. Phys. Rev. D.

[75] Bombelli L., Lee J., Meyer D., Sorkin R.D. (1987). Space-time as a causal set. Phys. Rev. Lett..

[76] Srednicki M. (1993). Entropy and area. Phys. Rev. Lett..

[77] Calabrese P., Cardy J. (2006). Entanglement entropy and quantum field theory: A non-technical introduction. Int. J. Quantum Inf..

[78] Nishioka T., Ryu S., Takayanagi T. (2009). Holographic entanglement entropy: An overview. J. Phys. A.

[79] Martín-Martínez E., Menicucci N.C. (2012). Cosmological quantum entanglement. Class. Quant. Grav..

[80] Martín-Martínez E., Menicucci N.C. (2014). Entanglement in curved spacetimes and cosmology. Class. Quant. Grav..

[81] Martín-Martínez E., Smith A.R., Terno D.R. (2016). Spacetime structure and vacuum entanglement. Phys. Rev. D.

[82] Martin J., Vennin V. (2021). Real-space entanglement in the cosmic microwave background. arXiv.

[83] Lin S.-Y., Chou C.-H., Hu B.L. (2016). Entanglement dynamics of detectors in an Einstein cylinder. JHEP.

[84] http://www.isrqi.net/.

[85] Kluger Y., Mottola E., Eisenberg J.M. (1998). Quantum Vlasov equation and its Markov limit. Phys. Rev. D.

[86] Koks D., Matacz A., Hu B.L. (1997). Entropy and uncertainty of squeezed quantum open systems. Phys. Rev. D.

[87] Dodonov V. (2020). Fifty years of the dynamical Casimir effect. Physics.

[88] Calzetta E.A., Hu B.L., Hu B.L., Jacobson T. (1993). Decoherence of correlation histories. Directions in General Relativity.

[89] Calzetta E.A., Hu B.L., Fulling S.A. (1995). Correlations, decoherence, dissipation, and noise in Quantum Field Theory. Proceedings of the International Workshop on Heat Kernel Techniques and Quantum Gravity.

[90] Zwanzig R. (2001). Nonequilibrium Statistical Mechanics.

[91] Hu B.L., D’Olivio J., Nahmad-Achar E., Rosenbaum M., Ryan M.P., Urrutia L.F., Zertuche F. (1991). Coarse-graining and backreaction in inflationary and minisuperspace cosmology. Relativity and Gravitation: Classical and Quantum.

[92] Johnson P.R., Hu B.L. (2000). Stochastic theory of relativistic particles moving in a quantum field: I. Influence functional and Langevin equation. arXiv.

[93] Calzetta E.A., Hu B.L., Mazzitelli F.D. (2001). Coarse-grained effective action and renormalization group theory in semiclassical gravity and cosmology. Phys. Rep..

[94] Johnson P.R., Hu B.L. (2002). Stochastic theory of relativistic particles moving in a quantum field: Scalar Abraham-Lorentz-Dirac-Langevin equation, radiation reaction, and vacuum fluctuations. Phys. Rev. D.

[95] Zhang Y. (1990). Stochastic Properties of Interacting Quantum Fields. Ph.D. Thesis.

[96] Hu B.L., Kobes R., Kunstatter G. (1994). Quantum statistical field theory in gravitation and cosmology. Proceedings of the Canadian Summer School for Theoretical Physics and the Third International Workshop on Thermal Field Theories and Applications.

[97] Boyanovsky D. (2016). Effective Field theory during in inflation II. Stochastic dynamics and power spectrum suppression. Phys. Rev. D.

[98] Prokopec T., Rigopoulos G.I. (2007). Decoherence from isocurvature perturbations in inflation. JCAP.

[99] Rai M., Boyanovsky D. (2020). Origin of entropy of gravitationally produced dark matter: The entanglement entropy. Phys. Rev. D.

[100] Nelson E. (2016). Quantum decoherence during inflation from gravitational nonlinearities. JCAP.

[101] Hollowood T.J., McDonald J.I. (2017). Decoherence, discord, and the quantum master equation for cosmological perturbations. Phys. Rev. D.

[102] Fukuma M., Sugishita S., Sakatani Y. (2014). Master equation for the Unruh-DeWitt detector and the universal relaxation time in de Sitter space. Phys. Rev. D.

[103] Polarski D., Starobinsky A.A. (1996). Semiclassicality and decoherence of cosmological perturbations. Class. Quant. Grav..

[104] Calzetta E.A., Hu B.L. (1997). Stochastic behavior of effective field theories across the threshold. Phys. Rev. D.

[105] Koksma J.F., Prokopec T., Schmidt M.G. (2012). Decoherence and dynamical entropy generation in quantum field theory. Phys. Lett. B.

[106] Kurki-Suonio H. Lecture Notes on Cosmological Perturbation Theory, Part 1. http://www.helsinki.fi/~hkurkisu/CosPer.pdf.

[107] Simon R., Mukunda N., Dutta B. (1994). Quantum-noise matrix for multimode systems: *U*(*n*) invariance, squeezing, and normal forms. Phys. Rev. A.

[108] Hsiang J.-T., Hu B.L. (2021). Nonequilibrium quantum free energy and effective temperature, generating functional, and influence action. Phys. Rev. D.

[109] Hsiang J.-T., Hu B.L. (2021). Fluctuation-dissipation relation for a quantum Brownian oscillator in a parametrically squeezed thermal field. Ann. Phys..

[110] Ford L.H. (2021). Cosmological particle production: A review. Rep. Prog. Phys..

[111] Sudarshan E.C.G. (1963). Equivalence of semiclassical and quantum mechanical descriptions of statistical light beams. Phys. Rev. Lett..

[112] Cahill K.E., Glauber R.J. (1969). Ordered expansions in boson amplitude operators. Phys. Rev..

[113] Agarwal G.S., Wolf E. (1970). Calculus for functions of noncommuting operators and general phase-space methods in quantum mechanics. II. quantum mechanics in phase space. Phys. Rev. D.

[114] Hsiang J.-T., Hu B.L. (2018). Quantum thermodynamics from the nonequilibrium dynamics of open systems: Energy, heat capacity, and the third law. Phys. Rev. E.

[115] Serafini A., Illuminati F., De Siena S. (2003). Symplectic invariants, entropic measures and correlations of Gaussian states. J. Phys. B.

[116] Hsiang J.-T., Hu B.L., Lin S.-Y. (2019). Fluctuation-dissipation and correlation-propagation relations from the nonequilibrium dynamics of detector-quantum field systems. Phys. Rev. D.

[117] Hsiang J.-T., Hu B.L., Lin S.-Y., Yamamoto K. (2019). Fluctuation-dissipation and correlation-propagation relations in (1+3)D moving detector-quantum field systems. Phys. Lett. B.

[118] Hsiang J.-T., Hu B.L. (2015). Distance and coupling dependence of entanglement in the presence of a quantum field. Phys. Rev. D.

[119] Hsiang J.-T., Hu B.L. (2018). Thermodynamics of quantum systems strongly coupled to a heat bath I. Operator thermodynamic functions and relations. Entropy.

